# Model Cell Lines and Tissues of Different HGSOC Subtypes Differ in Local Estrogen Biosynthesis

**DOI:** 10.3390/cancers14112583

**Published:** 2022-05-24

**Authors:** Renata Pavlič, Marija Gjorgoska, Tea Lanišnik Rižner

**Affiliations:** Institute of Biochemistry and Molecular Genetics, Faculty of Medicine, University of Ljubljana, 1000 Ljubljana, Slovenia; renata.pavlic@mf.uni-lj.si (R.P.); marija.gjorgoska@mf.uni-lj.si (M.G.)

**Keywords:** ovarian cancer, high-grade serous ovarian carcinoma, HIO-80, OVSAHO, Kuramochi, COV362, immunoreactive, differentiated, proliferative, mesenchymal subtype

## Abstract

**Simple Summary:**

Ovarian cancer (OC) comprises a heterogeneous group of hormone-dependent diseases with very high mortality. Estrogens have been shown to promote the progression of OC; however, their exact role in OC subtypes remains unknown. Here, we investigated the local estrogen biosynthesis in OC. We performed targeted transcriptomics and estrogen metabolism analyses in high-grade serous OC (HGSOC) cell lines that differed in chemoresistance status and compared these data with publicly available transcriptome and proteome data for HGSOC tissues. In HGSOC cells, estrogen metabolism decreased with increasing chemoresistance. In highly chemoresistant cells and platinum-resistant HGSOC tissues, *HSD17B14* expression was increased. Proteome data showed differential levels of HSD17B10, SULT1E1, CYP1B1, and NQO1 between the four HGSOC subtypes. Our results confirm that estrogen biosynthesis differs between different HGSOC cell models and possibly between different HGSOC subtypes. Such differentially expressed enzymes have potential as targets in the search of new treatment options.

**Abstract:**

Ovarian cancer (OC) is highly lethal and heterogeneous. Several hormones are involved in OC etiology including estrogens; however, their role in OC is not completely understood. Here, we performed targeted transcriptomics and estrogen metabolism analyses in high-grade serous OC (HGSOC), OVSAHO, Kuramochi, COV632, and immortalized normal ovarian epithelial HIO-80 cells. We compared these data with public transcriptome and proteome data for the HGSOC tissues. In all model systems, high steroid sulfatase expression and weak/undetected aromatase (*CYP19A1*) expression indicated the formation of estrogens from the precursor estrone-sulfate (E1-S). In OC cells, the metabolism of E1-S to estradiol was the highest in OVSAHO, followed by Kuramochi and COV362 cells, and decreased with increasing chemoresistance. In addition, higher *HSD17B14* and *CYP1A2* expressions were observed in highly chemoresistant COV362 cells and platinum-resistant tissues compared to those in HIO-80 cells and platinum-sensitive tissues. The HGSOC cell models differed in *HSD17B10*, *CYP1B1*, and *NQO1* expression. Proteomic data also showed different levels of HSD17B10, CYP1B1, NQO1, and SULT1E1 between the four HGSOC subtypes. These results suggest that different HGSOC subtypes form different levels of estrogens and their metabolites and that the estrogen-biosynthesis-associated targets should be further studied for the development of personalized treatment.

## 1. Introduction

Ovarian cancer (OC) is the seventh most common cancer in women worldwide [1] and has a very high mortality due to its asymptomatic nature, which usually results in the discovery of the disease in its advanced stages [2]. The survival of OC patients closely correlates with disease progression; the average 5-year survival rate of OC patients is only 47% [2].

More than 95% of all OCs are epithelial (EOC) [3]. EOC is an extremely heterogeneous group of malignant diseases that differ in clinicopathological features and genomic profiles [4]. Classified according to histological characteristics, the five most common forms of EOC are high-grade serous carcinoma (HGSOC; 70%), endometrioid carcinoma (10%), clear-cell carcinoma (5–10%), low-grade serous carcinoma (<5%), and mucinous carcinoma (2–3%) [4]. These subtypes share an anatomical location but are believed to be derived from different tissues. For example, HGSOCs are suggested to originate in the distal fallopian tube, and clear cell and endometrioid cancers have been associated with endometriotic lesions. Mucinous cancers usually present metastases to the ovary, often originating from the gastrointestinal tract [5].

Different OC subtypes have been traditionally classified as genetically stable type I or genetically unstable type II tumors. Recently, different OC subtypes have been preferentially classified based on their molecular characteristics [6]. For the most common OC, HGSOC, four robust subtypes have been discovered: immunoreactive, proliferative, differentiated, and mesenchymal subtypes, characterized by the expression of chemokines, proliferation markers, ovarian tumor markers, and markers suggestive of increased stromal components, respectively [7].

The development of OC can be explained by multiple hypotheses including the actions of gonadotropins, estrogens, androgens, and inflammatory molecules [8]. Studies have shown that estrogens promote OC development by increasing the proliferation of ovarian surface epithelial cells [9], inducing invasiveness [10], and increasing metastatic potential, as shown in some human epithelial cancer cell lines [11]. OC patients have increased estradiol plasma concentrations compared to the controls [12], and high activities of certain steroid-converting enzymes have been shown in OC tissues [9]. Both nuclear estrogen receptors (ER) α and β are expressed in most OCs [13], and the ERα/β ratio is usually elevated [14]. The literature on the association of ERα and the disease outcome or cisplatin-sensitivity of OC is opposing [15,16,17], with most studies associating ERα positivity with a good prognosis of serous OC patients [17]. ERβ expression is associated with antitumoral effects such as decreased growth rate, motility, and increased apoptosis, as observed in OC cells [18]. Additionally, the activation of ERβ with selective agonist results in the increased effectiveness of the cisplatin and paclitaxel treatment of OC cells [19].

In hormone-dependent cancers and local (extragonadal) tissues, estrogens can be formed from the inactive steroid precursor estrone sulfate (E1-S) via the sulfatase pathway (with steroid sulfatase (STS) as the key enzyme) or from dehydroepiandrosterone sulfate (DHEA-S) or androstenedione via the aromatase pathway (with aromatase (CYP19A1) as the key enzyme. The cellular uptake of precursors is enabled by organic anion transporting polypeptides (*SLCOs*, OATPs) and organic anion transporters (*SLCs*, OATs), whereas the efflux is enabled by ATP-binding cassette transporters (ABCs) and the organic solute transporter (OST) αβ (*SLC51*). Estrogen metabolism includes phase I, during which estrogens are hydroxylated by cytochrome P450 (CYP) enzymes, and phase II, during which catechol estrogens are conjugated by the actions of catechol-O-methyl transferase (COMT), sulfotransferases, uridine diphosphate (UDP)-glucuronosyltransferase, or estrogen quinones, which are inactivated by glutathione S-transferase (GSTP1) and NAD(P)H quinone dehydrogenases [20,21].

OC treatment usually involves cytoreductive surgery combined with platinum and taxane chemotherapy [22]. Although 60–80% of patients respond positively to such treatment, the disease relapses in most cases and becomes resistant to additional therapies [23]. Several mechanisms are involved in the development of OC resistance to chemotherapeutic agents including altered drug transport, which involves ABC transporters and altered cellular enzymes that are involved in detoxification (e.g., glutathione S transferases and aldo-keto reductases) [23,24].

For several years, estrogens have been associated with OC development and progression; however, no systematic study has investigated the expression of E1-S or DHEA-S transporters, estrogen biosynthetic or metabolic enzymes, or estrogen receptors in different HGSOC cell models or HGSOC subtype tissues. In this study, we used human OC cell lines from the most common OC type (HGSOC): OVSAHO, Kuramochi, and COV362 cells, which differ in their resistance to platinum derivatives, invasiveness, migratory ability, and proliferation [25]. We also used the normal ovarian epithelial cell line HIO-80 and the publicly available transcriptome and proteome data from the HGSOC tissues of different subtypes and normal fallopian tube tissue. In the cell lines, we evaluated the steroid precursor metabolism and performed a targeted transcriptomics study focusing on 50 key genes involved in steroid precursor transport and local estrogen biosynthesis. In the tissue samples, we evaluated the expression of these enzymes at the mRNA and protein levels. We hypothesized that differentially chemoresistant HGSOC cell lines and the tissues of different subtypes differ in their expressions of the transporters and enzymes involved in local estrogen biosynthesis and metabolism.

## 2. Materials and Methods

### 2.1. Cell Lines

The HIO-80 (CVCL_E274) immortalized normal ovarian epithelial cells were originally established from ovarian surface epithelium [26] and were obtained from Andrew K. Godwin (University of Kansas Medical Center, USA) as p72 on 20 October 2017. HIO-80 cells were grown in a 1:1 mixture of Medium 199 (M5017; Sigma-Aldrich GmbH) and MCDB105 medium (M6395) supplemented with 4% FBS (F9665) and 7.5 µg/mL insulin (I9278). HIO-80 cells from passages +10 to +20 were used in this study. HIO-80 cells in passage +10 were authenticated by short tandem repeat (STR) profiling performed by ATCC on 22 February 2019.

The OVSAHO (CVCL_3144) cell line was originally established from a serous papillary adenocarcinoma from a metastatic site in the abdomen [27] of a 56-year-old woman and was purchased from JCRB (JCRB1046 lot 04062015) as p44 on 4 June 2018. The growth medium for OVSAHO cells was RPMI (R5886; Sigma-Aldrich GmbH), with 10% FBS (F9665) and 2 mM L-glutamine (G7153). OVSAHO cells from passages +7 and +15 were used in this study. Authentication by STR profiling was performed by JCRB.

The Kuramochi (CVCL_1345) cell line was originally established from high-grade ovarian serous adenocarcinoma from a metastatic site in the ascites [28] and was purchased from JCRB (JCRB0098 lot 06302015) as p17 on 23 October 2017. The growth medium for Kuramochi cells was RPMI (R5886; Sigma-Aldrich GmbH), with 10% FBS (F9665) and 2 mM L-glutamine (G7153). Kuramochi cells from passages +5 to +15 were used in this study. Authentication by STR profiling was performed by JCRB.

The COV362 (CVCL_2420) cell line was originally established from a high-grade ovarian serous adenocarcinoma derived from a metastatic site in pleural effusion [29] and was purchased from ECACC (ECACC 07071910) as p37 on 13 October 2017. The growth medium for the COV362 cells was DMEM (D5546; Sigma-Aldrich GmbH), with 10% FBS (F9665) and 2 mM L-glutamine (G7153). COV362 cells from passages +8 to +15 were used in this study. Authentication by STR profiling was performed by ECACC.

All cell lines were negative for mycoplasma infection, which was periodically tested using the MycoAlert^TM^ Mycoplasma Detection Kit (Lonza, Basel, Switzerland).

### 2.2. RNA Isolation

The total RNA from the HIO-80, OVSAHO, Kuramochi, and COV362 cells was isolated and purified using Nucleospin RNA Isolation Kits (Macherey-Nagel GmbH & Co. KG, Düren, Germany), according to the manufacturer’s instructions. The RNA quantity and quality were determined using NanoDrop (Thermo Fisher Scientific, Waltham, MA, USA). The RNA was of very good quality, with an average RNA integrity number of 9.4 ± 0.39. Samples of the total RNA (4 µg) were reverse transcribed into cDNA (in 40 µg) using the SuperScript^®^ VILO™ cDNA Synthesis Kit (Invitrogen, Thermo Fisher Scientific, Carlsbad, CA, USA) according to the manufacturer’s instructions. The cDNA samples were stored at −20 °C. For RNA isolation, cell lines were cultured independently on three different occasions (n = 3).

### 2.3. Quantitative PCR

The expressions of the genes of interest (Table 1) were examined by quantitative PCR (qPCR) using the TaqMan^®^ Fast Advanced Master Mix (Applied Biosystems; Foster City, CA, USA) or SYBR Green I Master (Roche, Basel, Switzerland) as described in our previous studies [20]. Quantification was accomplished with the Applied Biosystems^®^ ViiA^TM^ 7 Real-Time PCR System (Thermo Fisher Scientific, Waltham, MA, USA). All cDNA samples were run in triplicate using 0.25 μL of cDNA, and the reactions were performed in Applied Biosystems^®^ MicroAmp^®^ Optical 384-well plates (Thermo Fisher Scientific, Waltham, MA, USA) in a reaction volume of 5.0 μL. The PCR amplification efficiency was determined from the slope of the log-linear portion of the calibration curve for each gene investigated, and this was accounted for in the further calculations. For the gene expression analysis, the normalization factor for each sample was calculated based on the geometric mean of the two most stably expressed reference genes (*POLR2A* and *RPLP0*). The gene expression for each sample was calculated from the crossing-point value (Cq) as *E*^−Cq^, divided by the normalization factor and multiplied by 10^12^. The Cq cutoff value was set to 35. The Minimum Information for Publication of Quantitative Real-Time PCR Experiments guidelines were considered in the performance and interpretation of the qPCR reactions [30].

### 2.4. Liquid Chromatography-Tandem Mass Spectrometry (LC-MS/MS)

HIO-80 (10–15th passages), OVSAHO (8–15th passages), Kuramochi (10–15th passages), and COV362 (8–15th passages) cells were plated into 6-well plates at cell densities of 1.1 × 10^5^, 2.0 × 10^6^, 5.0 × 10^5^, and 3.5 × 10^5^ cells/well, respectively. After 24 h, the cells were washed with DPBS, and serum-free and phenol-red-free culture medium was added. The cells were then incubated with 2.3, 8.5, and 85 nM E1-S (in ethanol with a final concentration of 0.25%). After 8, 24, 48, and 72 h of incubation, the cell culture medium was collected in microcentrifuge tubes (Eppendorf, Hamburg, Germany) and stored at −80 °C until further processing. Two independent experiments were carried out, each performed in duplicate.

E1-S metabolism in the cell lines was assessed using LC-MS/MS, which comprised a Shimadzu Nexera XR system (Shimadzu Corporation, Kyoto, Japan) and a triple quadruple MS system (Triple Quad 3 500; AB Sciex Deutchland GmbH, Darmstadt, Germany). The levels of E1-S, E1, E2, and E2-sulfate (E2-S) were assessed with the LC-MS/MS method as described previously [20]. Briefly, a deuterated internal standard of E2-d2 was added to the cell culture medium samples, and the lipophilic fraction containing the analytes of interest was extracted with solid-phase extraction (Strata-X polymer-based columns; Phenomenex, Torrance, CA, USA). The method involved column conditioning with 1 mL methanol, column equilibration with 1 mL water, sample loading, column drying at high vacuum for 10 min, and finally elution with 1.5 mL methanol. Next, the samples were evaporated under vacuum and reconstituted in 100 μL of 70% methanol/0.2 mM NH_4_F.

For chromatographic separation, we used a C18 column (Kinetex 2.6 μm XB; 100 × 4.6 mm; Phenomenex, Aschaffenburg, Germany). The mobile phase compositions included phase A (5% methanol in water, 0.2 mM NH4F) and phase B (100% methanol, 0.2 mM NH_4_F). The injection volume was 25 μL, and the oven temperature was 38 °C. The mobile phase flow rate was 0.5 mL/min, and the following gradient was used: 0.0–3.0 min, 70% A; 3.0–8.0 min, 70–4% A; 8.0–8.01 min, 4–70% A; 8.01–15.0 min, 70% A. The MS/MS analysis was performed in negative ion mode with constant electrospray ionization conditions. The source-dependent parameters were as follows: curtain gas, 50 psi; collision gas, 8 psi; ion spray voltage, −4500 V; source temperature, 600 °C; ion source gas 1, 40 psi; ion source gas 2, 80 psi. All transitions were recorded using the scheduled multiple reaction monitoring algorithm. The target scan time was set to 1 s, with a multiple reaction monitoring detection window of 120 s. The resolution for the first and third quadrupole (Q1, Q3) was set as the unit, and the pause between the mass ranges was set at 5 ms. The limits of detection and quantification were calculated as 3× and 10× the signal/noise ratio. The limit of detection for E1, E1-S, E2, and E2-S was 1 pg/mL. The limit of quantification was 5 pg/mL for E1 and E1-S and 10 pg/mL for E2 and E2-S.

### 2.5. Transcriptome and Proteome Analysis in HGSOC and Normal Tissues Using Publicly Available Databases

To analyze the gene expression in HGSOC tissues, we downloaded data from cBioPortal (https://www.cbioportal.org/) on 10 January 2022 [31,32]. For the analysis, we used the RNA expression data (acquired using the RNA-Seq by Expectation-Maximization algorithm, batch normalized) and clinical data from the Ovarian Serous Cystadenocarcinoma (TCGA, PanCancer Atlas) study.

To analyze the protein levels in the HGSOC and normal tissues, we accessed the National Cancer Institute, Proteomic Data Commons server (https://pdc.cancer.gov) on 12 January 2022. We used proteomic data from the TCGA Ovarian PNNL and JHU Proteome studies (IDs PDC000114, PDC000113) [33] and Prospective Ovarian JHU Proteome study (ID PDC000110) [34]. We used normalized log2 transformed levels of the proteins of interest from these studies.

### 2.6. Hierarchical Clustering

Hierarchical clustering was performed using RStudio, version 1.1.436 [35] with the ComplexHeatmap package [36]. We used log2 transformed gene expression data, Euclidean distance, and Ward’s linkage. The R code used for data clustering is presented in Supplementary Table S1.

### 2.7. Statistical Analysis

Statistical analysis was performed using GraphPad Prism software for Windows, version 8.0 (San Diego, CA, USA) and the Kruskal–Wallis with Dunn’s multiple comparisons, Mann–Whitney U, one-way ANOVA with Bonferroni multiple comparisons, or Tukey’s tests.

All data are shown as mean ± standard deviation (SD). Differences with *p* values < 0.05 were considered statistically significant.

## 3. Results and Discussion

### 3.1. Targeted Transcriptomic Analysis Suggests That Estrogen Metabolism Differs between Different HGSOC Cells

**Figure 1:**
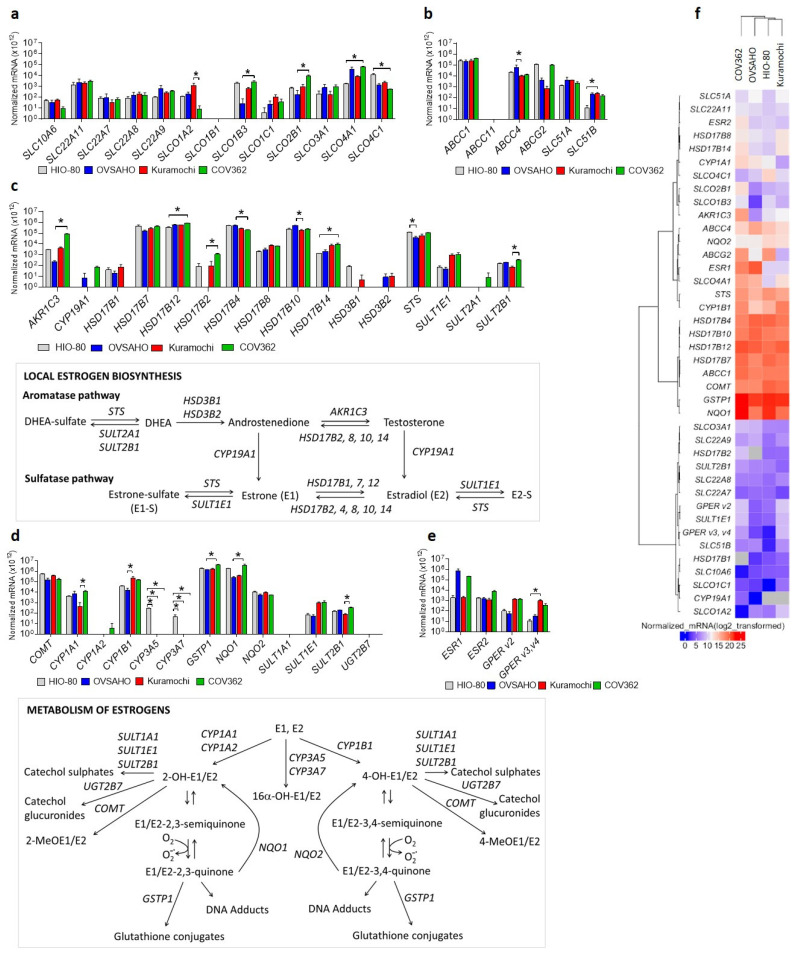
Gene expression of the (**a**) uptake transporters, (**b**) efflux transporters, (**c**) estrogen biosynthetic enzymes (with a schematic representation of local estrogen biosynthesis), (**d**) estrogen metabolic enzymes (with a schematic representation of estrogen metabolism), and (**e**) estrogen receptors in the HIO-80, OVSAHO, Kuramochi, and COV362 cell lines. (**f**) A heatmap with a dendrogram of the evaluated genes (excluding the weakly/not expressed genes *CYP1A2*, *CYP3A5*, *CYP3A7*, *HSD3B1*, *HSD3B2*, and *SULT2A1*) clustered based on Euclidean distance and Ward’s linkage. The expression of the genes of interest was evaluated in three individual experiments. Kruskal–Wallis with Dunn’s multiple comparison tests; *, *p* < 0.05. Data are presented as means ± SD. Normalized mRNA values for individual genes are shown in Supplementary Table S2.

We examined the expression (Cq < 35) of 50 genes: 13 uptake and five efflux transporters of steroid precursors, 16 biosynthetic, and 13 metabolic enzymes for estrogens, two nuclear receptors, and all three variants of the membrane estrogen receptor (Figure 1). We used the normal ovarian epithelial cell line HIO-80 and three different chemoresistant HGSOC cell lines: the least chemoresistant OVSAHO (cisplatin IC50~3.7 µM [25], carboplatin IC50 = 9.4 µM [37]), moderately chemoresistant Kuramochi (cisplatin IC50~10.4 µM [25], carboplatin IC50 = 12 µM [37]), and highly chemoresistant COV362 cell line (cisplatin IC50 = 13.57 µM [25], carboplatin IC50 = 318.2 µM [38]).

Among the transporters, *SLCO4A1* (uptake) and *ABCC1* (efflux) had the highest expression, and the differential expression was detected for all expressed *SLCOs* (uptake) and *ABCC4* (efflux). In all cell lines, aromatase (*CYP19A1*) expression was weak or undetectable, whereas the *STS* expression was high, indicating the importance of the sulfatase pathway for estrogen formation. Genes for the *HSD17B* enzymes were highly and differentially expressed between cells, indicating differences in estrogen metabolism. In all cell lines, *GSTP1* and *NQO1* expression significantly exceeded the expression of all the estrogen-hydroxylating *CYP* enzymes examined, indicating a high level of estrogen quinone detoxification. All estrogen receptors were expressed, with significantly more *ESR1* than the G protein-coupled estrogen receptor (*GPER) v3,v4* in HIO-80 (*ESR1/GPER v3,v4* = 171.3), OVSAHO (*ESR1/GPER v3,v4* = 23,173.5), and COV362 cells (*ESR1/GPER v3,v4* = 560.0). *ESR1* mRNA levels were the highest in the OVSAHO, followed by the COV362 and Kuramochi cells, and were in line with the reported protein levels, except for the Kuramochi cells, in which ERα was previously not detected [39]. Based on the gene expression data, the HIO-80 and Kuramochi cells are very similar, whereas the COV362 cells differed the most.

The genes evaluated in the current study are not only involved in estrogen metabolism, but also in the transport of anticancer drugs (e.g., *SLCs* and *SLCOs* [40], or indirectly in cell cycle regulation (e.g., *HSD17B7* [41]), proliferation (e.g., *GPER* [42]), apoptosis (e.g., *GSTP1* [43]), and other processes. Cell lines used in our study have different genetic backgrounds including alterations in the genes regulating the cell cycle, cell maturation, and apoptosis (e.g., *RB1*, *MYC*, *KRAS* [44]), possibly associated with their differences in proliferation, migration, and colony formation [25]. Despite the possibility that the differential expression of evaluated genes may arise due to several processes, in the continuation, we focus more on one aspect of this complex web of interactions: the metabolism of the less investigated estrogens.

### 3.2. Differential Gene Expression Correlates with Differences in Chemoresistance

**Figure 2:**
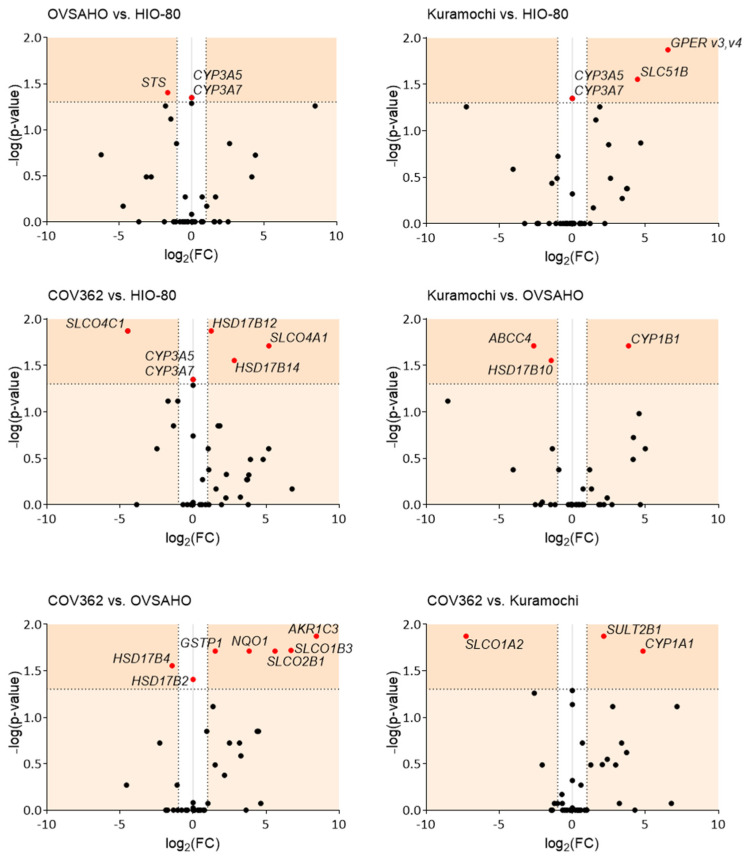
Pairwise comparison of the gene expression in the HIO-80, OVSAHO, Kuramochi, and COV362 cells, presented as volcano plots. FC, fold change; horizontal dashed line, the cutoff for experimental significance (dark orange; −log (1.3); *p* < 0.05); vertical dashed lines, the cutoff for genes similarly expressed in both cell lines (FC, ±2.0); vertical grey line (x = 0), genes not expressed in either cell line; red dots, differentially expressed genes; black dots, non-differentially expressed genes. Fold regulation and *p* values (Mann–Whitney U tests) of gene expression for individual cell pairs are presented in Supplementary Table S4.

We compared the cell lines pairwise based on gene expression (Figure 2). Compared to cells of normal ovarian epithelium, HIO-80, the smallest differences in gene expression were observed in OVSAHO, followed by the Kuramochi and COV362 cells. In OVSAHO cells, lower *STS* expression (3.1-fold) indicated a lower formation of E1 and DHEA from sulfated steroid precursors. In the Kuramochi cells, increased *GPER v3,v4* expression (93.3-fold) suggested a more important role of GPER-associated signaling and increased *SLC51B* expression (22.0-fold, Km for E1-S = 320 µM [45] (Supplementary Table S3)), indicating greater formation of the dimeric OSTαβ efflux transporter [46]. In the COV362 cells, we observed an increase in *SLCO4A1* (36.5-fold) and *HSD17B14* (7.1-fold) and a decrease in *SLCO4C1* (22.1-fold, Km for E1-S = 27 µM [45]) and *HSD17B12* (2.4-fold), indicating differences in the uptake of steroid precursors and E2 formation compared to the HIO-80 cells. None of the HGSOC cell lines expressed *CYP3A5* or *CYP3A7,* suggesting that estrogens cannot be 16α-hydroxylated in these cells.

Compared to the least chemoresistant OVSAHO cells, the Kuramochi cells expressed less *ABCC4* (6.2-fold) and *HSD17B10* (2.7-fold) and more *CYP1B1* (14.4-fold), indicating less steroid precursor efflux, less E2 deactivation to E1, and more 4-OH-E1/E2 formation in the Kuramochi cells. Conversely, compared to the OVSAHO cells, highly chemoresistant COV362 cells expressed higher levels of *SLCO1B3* (104.4-fold, Km for E1-S = 5–58 µM [45]) and *SLCO2B1* (48.8-fold, Km for E1-S = 1.6–21 µM [45]), indicating more uptake of steroid precursors, and lower levels of *HSD17B4* (2.7-fold) and higher levels of *HSD17B2* (which was not expressed in OVSAHO cells), indicating higher deactivation of E2 to E1 in the COV362 cells. Additionally, in the COV362 compared to OVSAHO cells, the levels of the following genes were higher: *AKR1C3* (348.2-fold), indicating more conversion of androstenedione to testosterone, and *NQO1* (14.4-fold) and *GSTP1* (2.9-fold), indicating more inactivation of estrogen-quinones in the COV362 cells. In the COV362 cells compared to Kuramochi cells, increased *SULT2B1* (4.5-fold) and *CYP1A1* (28.6-fold) and decreased *SLCO1A2* (155.0-fold) levels were observed, indicating increased formation of catechol sulfates and sulfated DHEA and decreased uptake of steroid precursors, respectively, in the COV362 cells.

Some of the differentially expressed genes were previously associated with chemoresistance, prognosis, and cell proliferation. For example, the expression levels of *AKR1C3*, an enzyme that has a role in chemoresistance establishment [24], are higher in COV362 than in OVSAHO cells. Furthermore, several *SLCOs* were upregulated in HGSOC tissues compared to benign ovarian cysts [47], and *SLCO1B1* and *SLCO1B3* promoted paclitaxel uptake [48]. In our study, several *SLCO* transporters were differentially expressed in COV362 compared to the less chemoresistant cells or normal ovarian cells. As COV362 cells are more chemoresistant to paclitaxel (IC50 = 2.72 nM [49]) compared to the Kuramochi (IC50 = 1.51 nM [49]) and OVSAHO cells (IC50 = 0.276 nM [49]), the different transporter profile in highly chemoresistant HGSOC indicates associations with processes other than drug transport, perhaps with the functional characteristics of the COV362 cells or the increased energy demands of cancer cells [50]. Furthermore, studies have also shown that paclitaxel-induced CYP1B1 expression causes OC drug resistance [51] and that CYP1A1 contributes to OC initiation and progression [52]. In our study, we observed increased *CYP1B1* levels in the Kuramochi compared to OVSAHO cells and increased the *CYP1A1* levels in COV362 compared to the Kuramochi cells, which indicates an association with the prognosis of these HGSOC models.

In OC, ABC transporters were associated with aggressiveness (ABCC1) [53], unfavorable outcome (ABCC4) [53], and progression (ABCG2) [54]. Interestingly, we observed decreased *ABCC4* levels in Kuramochi compared to OVSAHO cells, which may be associated with lower proliferation rates of Kuramochi cells [25], as observed in non-small cell lung cancer cells [55]. Additionally, compared to the Kuramochi cells, slightly more proliferative COV362 cells [25] expressed more *SULT2B1*, which may also be associated with proliferation, as shown in hepatocellular carcinoma cells [56]. In the literature, the data on GPER are opposing; however, numerous studies have associated GPER with the mechanisms of carcinogenesis [57]. Our results suggest that GPER is associated with cancer progression because the *GPER v3,v4* mRNA levels were higher in the Kuramochi than those in normal HIO-80 cells. The importance of GPER has already been shown in ERα-negative/GPER-positive OVCAR5 OC cells in which E2 induced cell proliferation [42]. This could also be the case for Kuramochi cells because these cells do not express ERα [39].

Overall, our results indicate the association of multiple transporters and estrogen metabolic enzymes with the severity of HGSOC. Specifically, we observed an increasing number of differentially expressed genes with increasing chemoresistance of OC cells, indicating an association between estrogen metabolism and chemoresistance.

### 3.3. OVSAHO Cells Have the Highest Capacity for E1-S Uptake and Metabolism

To evaluate the differences in estrogen metabolism between the normal and HGSOC cell lines, we used 2.3 nM, 8.5 nM, and 85 nM E1-S and measured the products E1, E2, and E2-S with LC-MS/MS 24, 48, and 72 h after treatment.

**Figure 3:**
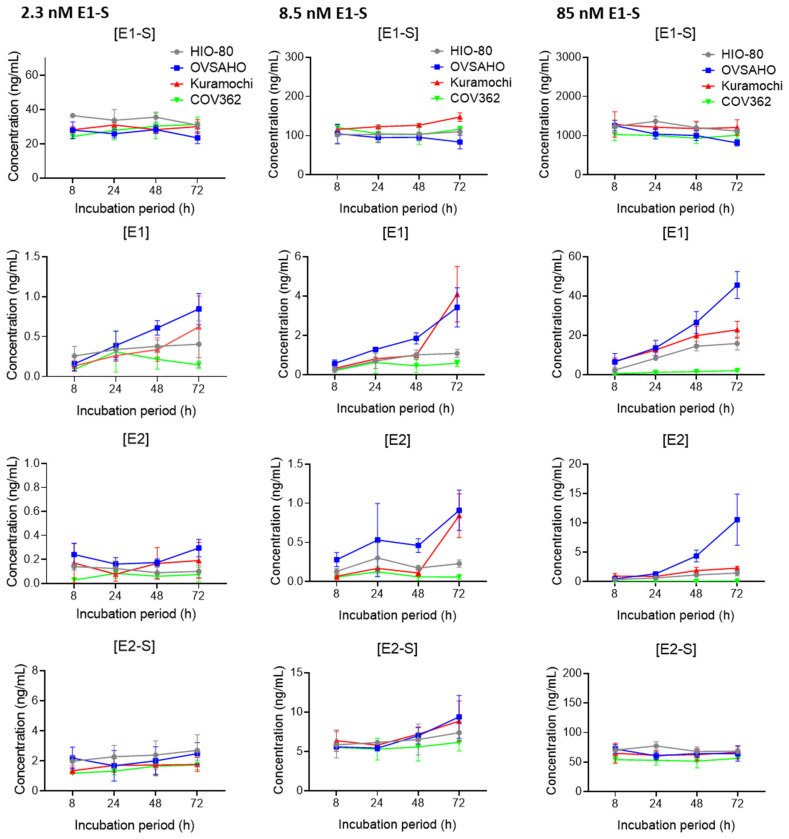
E1-S metabolism in HIO-80, OVSAHO, Kuramochi, and COV362 cells. Time courses for the estrogen metabolites E1-S, E1, E2, and E2-S following the addition of 2.3 nM E1-S (**left**), 8.5 nM E1-S (**middle**), or 85 nM E1-S (**right**) to the cells. Data are presented as means ± SD of two individual experiments. Several statistically significant differences (Mann–Whitney U test) in the gene expression are presented in Supplementary Table S5. Values on the graphs are presented in Supplementary Table S6.

The formation of E1 and E2 was the fastest and largest in the OVSAHO cells (Figure 3, Supplementary Table S5), followed by the Kuramochi, HIO-80, and COV362 cells. At lower E1-S concentrations, the Kuramochi and HIO-80 cells showed a similar capacity for E1-S uptake and metabolism, and at the highest E1-S concentration, the Kuramochi cells formed more E1 than the HIO-80 cells. In the COV362 cells, the formation of E1 was minimal, and the formation of E2 was undetectable.

Our results confirmed E2 formation via the sulfatase pathway in the OVSAHO, Kuramochi, and HIO-80, but not in the COV362 cells. In the COV362 cells, E2 formation may be limited by an altered *HSD17B* enzyme ratio. The most potent enzyme for the transformation of E1 to E2, *HSD17B1* (Km = 0.9 µM [58]), was undetectable in the COV362 cells, whereas the most potent enzyme for the transformation of E2 to E1, *HSD17B2* (Km = 0.21 µM [59]), was highly expressed in COV362 and undetectable in the OVSAHO cells. The resulting high *HSD17B1/HSD17B2* ratio in the OVSAHO cells and low ratio in the COV362 cells could explain the strong E2 formation in the OVSAHO cells and the lack of E2 formation in the COV362 cells.

Lower formation of estrogens observed in the Kuramochi and COV362 cells compared to the OVSAHO cells may, to some extent, be associated with the differences in the oxidative metabolism of estrogens in these cells. Gene expression data indicate that, compared to the OVSAHO cells, the Kuramochi cells transform E1-S in higher levels to catechols (decreased *ABCC4* and *HSD17B10* and increased *CYP1B1*), and COV362 cells form higher levels of 2-OH-E1/E2 (increased *NQO1*) and glutathione-conjugated quinones (increased *GSTP1*). Additionally, in the COV362 cells compared to the Kuramochi cells, higher catechols (increased *CYP1A1*) and catechol sulfates (increased *SULT2B1)* are expected, supporting the lower E1-S metabolism seen in the COV362 cells. The exact differences in the levels of oxidative metabolites and their conjugates are currently unknown and are yet to be determined.

In highly chemoresistant COV362 cells and moderately chemoresistant Kuramochi cells, *HSD17B* enzymes with lower efficiency for estrogen transformation, *HSD17B12* (Km for E1 = 3.5 µM [60]), *HSD17B14* (Km for E2 = 5.6 µM [61]), and *HSD17B10*, were also differentially expressed compared to the HIO-80 and OVSAHO cells, respectively (Figure 2). The expression patterns of these genes were not in line with the observed E2 formation, which indicates that other processes catalyzed by the enzymes *HSD17B12*, *HSD17B14*, and *HSD17B10* may also be crucial for OC progression. In accordance with this finding, a previous study demonstrated that HSD17B12 might be associated with increased prostaglandin formation in OC progression [62], but our results here indicate that these HSD17B enzymes may also be associated with chemoresistance.

The limiting step for estrogen biosynthesis in COV362 cells is also the uptake and transformation of E1-S to E1, as only minimal E1 formation was observed at the highest E1-S concentration in the COV362 cells. The lack of E1 formation is not in line with the increased *SLCO1B3* (Km for E1-S = 5–58 µM [45]) and *SLCO2B1* levels (Km for E1-S = 1.6–21 µM [45]) in COV362 compared to the OVSAHO cells, but can be partially explained by lower *STS* expression (*STS/SULT1E1* ratios: HIO-80, 1695.2; OVSAHO, 742.5; Kuramochi, 64.8; COV362, 101.3). Increased *SLCO1B3* and *SLCO2B1* could indicate the potential importance of DHEA-S and the formation of androgens in these cells, which is also indicated by an increase in *AKR1C3* expression.

Altogether, the gene expression differences in the highly chemoresistant COV362 cells compared to the least chemoresistant OVSAHO cells are concordant with the low/undetectable formation of E1 and E2 from E1-S in the COV362 cells and show an association with lower *STS/SULT1E1* ratio, the lack of *HSD17B1* expression, and potentially higher formation of oxidative metabolites and their conjugated metabolites in these cells.

Estrogens most likely play a more important role in OVSAHO cells because these cells are responsive to estradiol when grown as 3D spheroids, whereas COV362 cells are not [39]. Additionally, OVSAHO cells are the least chemoresistant of the evaluated cells, and studies have shown that carboplatin-sensitive cells produce more steroid hormones and metabolites than platinum-resistant cells [37]. Nevertheless, the lack of E1-S metabolism cannot exclude the possibility of the paracrine or endocrine roles of estrogens in the COV362 cells, considering that *ESR1* expression at the mRNA level is relatively high, and ERα expression at the protein level is moderate (compared to the OVSAHO and Kuramochi cells) [39]. Furthermore, studies have shown that the tumor volume of COV362 xenografts in immunocompromised NOD scid gamma (NSG) mice increases in the presence of E2, and, once established, the tumor grows steadily [39]. Our results here show that if the growth of COV362 cells is estrogen-dependent, E2 cannot be formed from E1-S, and most probably neither from E1 nor DHEA-S.

### 3.4. Gene Expression in Tissues Is in Line with Poor Prognosis of HGSOC

**Figure 4:**
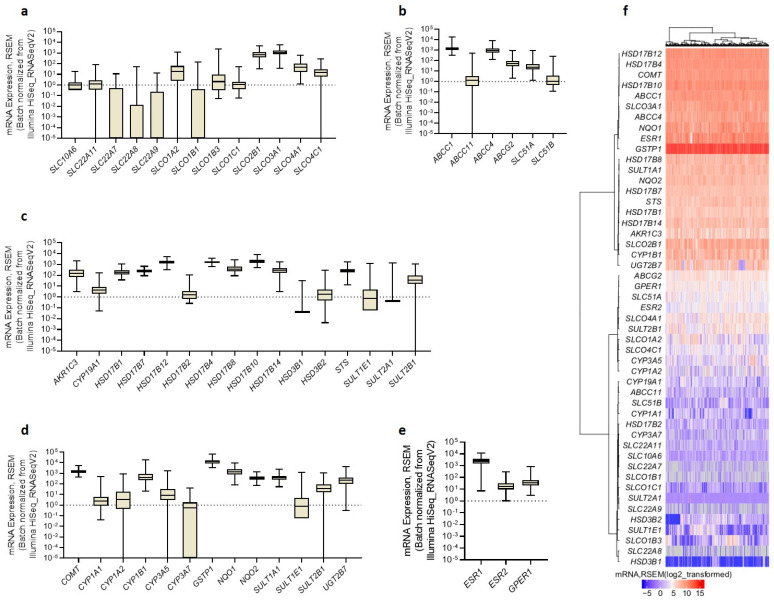
The expression of genes for (**a**) uptake transporters, (**b**) efflux transporters, (**c**) estrogen biosynthetic enzymes, (**d**) estrogen metabolic enzymes, and (**e**) estrogen receptors in the HGSOC tissues. (**f**) A heatmap with a dendrogram of all evaluated genes clustered based on the Euclidean distance and Ward’s linkage. The data from the Ovarian Serous Cystadenocarcinoma (TCGA, PanCancer Atlas) study were downloaded from cBioPortal on 10 January 2022. Data are presented as means ± SD (n = 300). Statistically significant differences (One-way ANOVA with Bonferroni correction) are shown in Supplementary Table S7.

To examine whether cell gene expression correlates with expression in HGSOC tissues, we used publicly available datasets at cBioPortal (https://www.cbioportal.org/, accessed on 10 January 2022) (Figure 4).

In the HGSOC tissues, we observed a high expression of *SLCO3A1* and *SLCO2B1*, followed by *SLCO4A1*, *SLCO4C1*, *SLCO1A2*, and *SLCO1B3*, and weak expression of *SLCO1C1*, *SLCO1B1*, and all of the evaluated *SLC* transporters. Of the efflux transporters, we observed a high expression of *ABCC1*, followed by *ABCC4* and *ABCG2*, and low expression of *ABCC11* and *SLC51B.* The expression of *STS* exceeded that of *CYP19A1* by approximately 36-fold, and the expression of *HSD3B1* and *HSD3B2* was weak. This suggests that the aromatase pathway is not involved in estrogen formation, whereas the sulfatase pathway may play a pivotal role. The expression of *SULT1E1* was weak, and the *STS/SULT1E1* ratio was 15.8, which indicates that E1-S is preferentially activated to E1 in HGSOC tissues. The expression of *AKR1C3* and *HSD17Bs* was relatively high. The mean mRNA levels of *HSD17B10*, *HSD17B12*, and *HSD17B4* significantly exceeded those of *HSD17B1*, *HSD17B14*, *HSD17B7*, and *HSD17B8* (Supplementary Table S7). The detoxification phase II enzymes *GSTP1, NQO1,* and *COMT* significantly surmounted the expression of all *CYP* genes coding for estrogen-hydroxylating enzymes. Of the estrogen receptors, *ESR1* expression was the highest, and significantly higher than that of *ESR2* and *GPER1* (by 104-fold and 58-fold, respectively).

Expression trends of the evaluated genes were generally similar in the HGSOC tissues and cell lines and in line with poor prognoses. In OC with poor prognosis, high *STS* and low *SULT1E1* expression are expected [63,64,65], and high *AKR1C3* expression is associated with higher chemoresistance [24]. Additionally, the expression of detoxification phase II metabolic enzymes, which usually prevent the formation of OC-initiating DNA adducts [66], can be associated with unfavorable disease outcomes. High *NQO1* expression was associated with the poor prognosis of serous OC [67], and high *GSTP1* expression was associated with higher chemoresistance, invasiveness, and migratory capacity of OC cells [43]. ERβ (ESR2) acts as a tumor suppressor in ovarian tissue and is lost with the severity of malignant transformation, and thus low ESR2 levels are expected in HGSOC [68,69,70].

Several highly expressed genes in HGSOC tissues are associated with the poor prognosis of OC. The altered expression pattern of several of these genes may affect the prognosis of HGSOC subtypes by influencing estradiol formation, as observed in the HGSOC model cell lines.

### 3.5. HSD17B14 and CYP1A2 Are Associated with Chemoresistance in HGSOC Tissues

**Figure 5:**
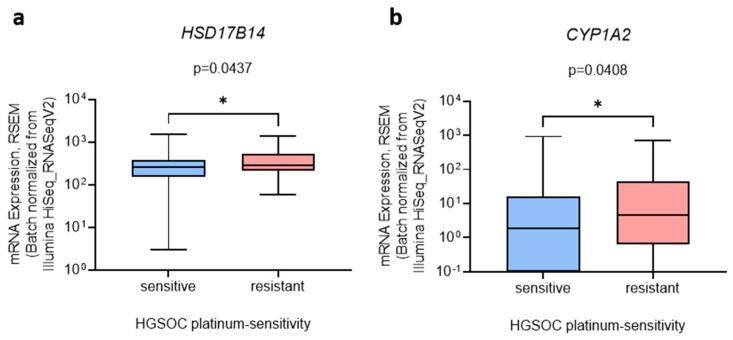
The expression of (**a**) *HSD17B14* and (**b**) *CYP1A2* in platinum-sensitive and platinum-resistant HGSOC tissues. Data from the Ovarian Serous Cystadenocarcinoma (TCGA, PanCancer Atlas) study were downloaded from cBioPortal on 10 January 2022. Data are presented as means ± SD (n (sensitive) = 105, n (resistant) = 43). Mann–Whitney U test; *, *p* < 0.05.

It has been proposed that differentially expressed genes are associated with prognosis, and thus we evaluated their association with HGSOC chemoresistance. *HSD17B14* and *CYP1A2* expression was slightly but significantly increased (by 1.3-fold and 1.7-fold, respectively) in the platinum-sensitive compared to platinum-resistant HSGOC (Figure 5).

A similar trend for *HSD17B14* and *CYP1A2* expression was also observed in the cell lines. *HSD17B14* expression was increased in highly chemoresistant COV362 cells compared to normal ovarian cells, HIO-80, and *CYP1A2* mRNA was detected only in the COV362 cells, indicating the importance of these genes in highly chemoresistant HGSOC. Regarding estrogen metabolism, high *HSD17B14* and *CYP1A2* expression may be associated with decreased E2 formation in more chemoresistant HGSOC cell models and may lead to the increased deactivation of E2 to E1 and increased the formation of harmful 2-OH-E1/E2, respectively. These effects are in accordance with our estrogen metabolism results, which showed that less E2 is formed in more chemoresistant HGSOC cells.

There is currently no data on the prognostic role of HSD17B14 in OC. However, in breast cancer, high *HSD17B14* expression is associated with improved recurrence-free and breast cancer-specific survival [71]. Additionally, high HSD17B14 protein levels in lymph node-negative ER+ breast cancer tumors indicate better outcomes of adjuvant tamoxifen treatment with fewer local recurrences [72]. There is little data regarding an association between *CYP1A2* expression and OC. *CYP1A2* polymorphisms are associated with an increased risk of OC in the Caucasian population [73,74]. However, no polymorphisms were reported for any cell lines or tissues evaluated in our study (cBioPortal, accessed on 9 February 2022). Furthermore, the analysis of *HSD17B14* and *CYP1A2* expression regarding the overall and disease-specific survival of resistant compared to sensitive HGSOC tumors did not show any correlations (not shown).

Our results indicate that differential expression of *HSD17B14* and *CYP1A2* is associated with a decrease in estrogen formation in highly chemoresistant HGSOC. However, further studies are needed to elucidate the exact role of *HSD17B14* and *CYP1A2*.

### 3.6. Protein Levels in Tissues Suggest Differences in Estrogen Biosynthesis between HGSOC Subtypes

To further validate our results at the protein level, we used publicly available datasets at the NCI, PDC server (https://pdc.cancer.gov, accessed on 12 January 2022). The levels of the proteins of interest were analyzed in two studies that contained proteomic data for OC. The first study (IDs PDC000114, PDC000113) included 169 HGSOC samples [33], and the second study (ID PDC000110) included 83 HGSOC samples and 23 normal fallopian tube tissue samples (13 samples were paired) [34]. The investigated studies used a non-targeted approach, so relative expression data of only the most abundant proteins were available.

**Figure 6:**
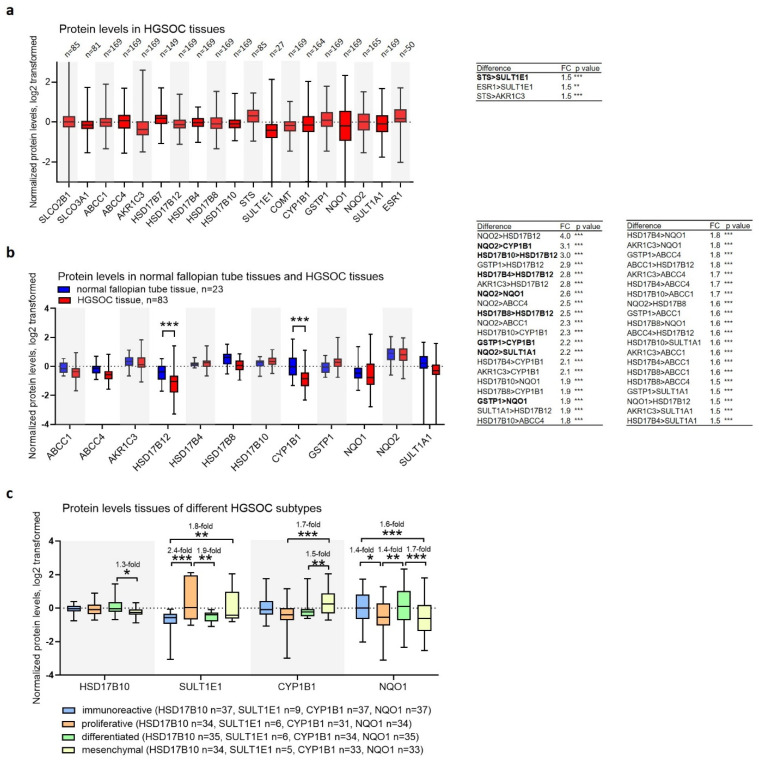
The normalized protein levels in (**a**) HGSOC (study IDs PDC000114, PDC000113) [33], (left) and significant differences between individual proteins (right), (**b**) normal fallopian tube and HGSOC tissues (study ID PDC000110 [34], left) and significant differences between individual proteins in the HGSOC tissues (right), (**c**) different subtypes of HGSOC (study IDs PDC000114, PDC000113) [33]). All the data were downloaded from the NCI, PDC server on 12 January 2022 and are shown as mean ± SD. One-way ANOVA with Bonferroni correction (**a**,**b**) and Tukey’s tests (**c**); *, *p* < 0.05; **, *p* < 0.01; ***, *p* < 0.001; FC, fold change; bold, differences that are more important for interpretation; »>« denotes »levels are higher than«.

The HGSOC tissues showed higher levels of STS than SULT1E1 (1.5-fold) and higher levels of HSD17B4 (3.0-fold), HSD17B8 (2.8-fold), and HSD17B10 (2.8-fold) when all were compared to HSD17B12, indicating an increased formation of E1 from E1-S and E2 (Figure 6). Compared to the normal fallopian tube tissues, the HSD17B12 and CYP1B1 levels were decreased (both by 1.8-fold), also indicating higher levels of E1 in the HGSOC compared to the fallopian tube tissues. The four HGSOC subtypes showed differential levels of HSD17B10, SULT1E1, CYP1B1, and NQO1, indicating differences in E2 biosynthesis and oxidative metabolism. In the HGSOC subtypes, the differential protein levels of transporters and enzymes indicated alterations in precursor transport, E1 and E2 formation, and estrogen metabolism (Supplementary Figure S1).

Our results indicate low E2 formation in the HGSOC tissues, as observed in the COV362 and Kuramochi cells. In addition, differences in CYP1B1 and NQO1 between different HGSOC subtypes were also observed in the cell line models differing in estrogen metabolism; compared to the OVSAHO cells, *CYP1B1* was increased in the Kuramochi cells, and *NQO1* was increased in the COV362 cells. Unfortunately, the subtypes of the investigated cell lines are currently unknown. Nevertheless, our results suggest that the four HGSOC subtypes may differ in estrogen metabolism due to differential expression of the enzymes HSD17B10, SULT1E1, CYP1B1, and NQO1.

In OC tissues, HSD17B12 levels have been shown to vary; lower levels were associated with better overall survival of the untreated OC patients [75]. Additionally, CYP1B1 levels were increased in primary and metastatic OC [76]. Normal ovarian epithelium did not express any of these proteins [75,76,77], whereas normal fallopian tube tissue expressed relatively high levels of CYP1B1 [77]. High CYP1B1 expression in the HGSOC and fallopian tube tissues may be explained by the theory of the fallopian tube origin of HGSOC. However, due to the lack of data on HSD17B12 and the other enzyme levels, the differences in estrogen metabolism between the HGSOC and fallopian tube tissues need to be further investigated.

## 4. Conclusions

Estrogens are associated with OC progression; however, their exact roles in the OC subtypes are unknown. To elucidate the role of estrogen formation in the subtypes of the most common OC, HGSOC, we performed targeted transcriptomics and estrogen metabolism analysis in the different cell lines of HGSOC and normal ovarian epithelium. Our results show that different HGSOC models indeed differ in estradiol formation and that differences in gene expression are associated with chemoresistance. Furthermore, comparing the results with public HGSOC tissue data revealed that chemoresistant tumors expressed more *HSD17B14* and *CYP1A2* than the chemosensitive cases. Of the evaluated cell lines, the COV362 cells expressed the highest *HSD17B14* and *CYP1A2* mRNA levels, indicating the importance of these enzymes in only highly chemoresistant HGSOC. In addition, the proteome data analysis showed lower HSD17B12 and CYP1B1 levels in HGSOC than those in the fallopian tube tissues, indicating a difference in the estrogen metabolism between HGSOC and the site of HGSOC origin. Furthermore, different HGSOC subtypes (i.e., immunoreactive, proliferative, differentiated, and mesenchymal) had different protein levels of HSD17B10, SULT1E1, CYP1B1, and NQO1, suggesting differences in the E2 formation between these subtypes. The limitations of our study were the lack of protein expression data for the less-expressed estrogen-metabolism-associated transporters and enzymes in the tissues and the lack of estrogen metabolism studies in HGSOC tissues. These call for future investigation.

Overall, the results confirmed our initial hypothesis: different HGSOC cases differ in their expressions of transporters and enzymes, which results in different estrogen metabolisms. Differential levels of individual enzymes between the HGSOC subtypes warrant further research toward the development of new treatment options.

## Figures and Tables

**Table 1:** The assays and sequences of the primers and probes to evaluate the genes of interest.

Gene	Gene Name	Assay	Assay ID or Sequences or Primers and/or Probes (5′ to 3′)
*ABCC1*	Multidrug-resistance-associated protein 1	SYBR Green	Forward primer: GGACTCAGGAGCACACGAAA
			Reverse primer: ACGGCGATCCCTTGTGAAAT
*ABCC11*	ATP-binding cassette subfamily C member 11	SYBR Green	Forward primer: TCTCCATATATCCTGTTAAT
			Reverse primer: TATAGTTCTCCAGTCTCTTG
*ABCC4*	Multidrug-resistance-associated protein 4	SYBR Green	Forward primer: AACTGCAACTTTCACGGATG
			Reverse primer: AATGACTTTTCCCAGGCGTA
*ABCG2*	Broad substrate specificity ATP-binding cassette transporter ABCG2	SYBR Green	Forward primer: GGGTTTGGAACTGTGGGTAG
			Reverse primer: AGATGATTCTGACGCACACC
*AKR1C3*	Aldo-keto reductase family 1, member C3 (17β-hydroxysteroid dehydrogenase type 5)	TaqMan	Forward primer: GTTGCCTATAGTGCTCTGGGATCT
			Reverse primer: GGACTGGGTC CTCCAAGAGG
			Fluorescent MGB-NFQ probe: CACCCATCGTTTGTCTC FAM
*COMT*	Catechol-O-methyltransferase	Taqman	Hs00241349_m1
*CYP19A1*	Cytochrome P450, family 19, subfamily A	Taqman	Hs00240671_m1
*CYP1A1*	Cytochrome P450, family 1, subfamily A, polypeptide 1	Taqman	Hs00153120_m1
*CYP1A2*	Cytochrome P450, family 1, subfamily A, polypeptide 2	Taqman	Hs00167927_m1
*CYP1B1*	Cytochrome P450, family 1, subfamily B, polypeptide 1	Taqman	Hs00164383_m1
*CYP3A5*	Cytochrome P450, family 3, subfamily A, polypeptide 5	Taqman	Hs00241417_m1
*CYP3A7*	Cytochrome P450, family 3, subfamily A, polypeptide 7	Taqman	Hs00426361_m1
*ESR1*	Estrogen receptor 1 (α)	TaqMan	Hs00174860_m1
*ESR2*	Estrogen receptor 2 (β)	TaqMan	Hs00230957_m1
*GPER v2*	G-protein–coupled estrogen receptor 1 (gene variant 2)	Taqman	Hs00173506_m1
*GPER v3*, *v4*	G-protein–coupled estrogen receptor 1 (gene variants 3 and 4)	Taqman	Hs01116133_m1
*GSTP1*	Glutathione S-transferase pi 1	Taqman	Hs00168310_m1
*HSD17B1*	Hydroxysteroid (17β) dehydrogenase 1	TaqMan	Hs00166219_g1
*HSD17B10*	Hydroxysteroid (17β) dehydrogenase 10	TaqMan	Hs00189576_m1
*HSD17B12*	Hydroxysteroid (17β) dehydrogenase 12	TaqMan	Hs00275054_m1
*HSD17B14*	Hydroxysteroid (17β) dehydrogenase 14	Taqman	Hs00212233_m1
*HSD17B2*	Hydroxysteroid (17β) dehydrogenase 2	TaqMan	Hs00157993_m1
*HSD17B4*	Hydroxysteroid (17β) dehydrogenase 4	TaqMan	Hs00264973_m1
*HSD17B7*	Hydroxysteroid (17β) dehydrogenase 7	Taqman	Hs00367686_m1
*HSD17B8*	Hydroxysteroid (17β) dehydrogenase 8	TaqMan	Hs00367151_m1
*HSD3B1*	Hydroxy-delta-5-steroid dehydrogenase, 3β, and steroid delta-isomerase 1	Taqman	Hs00426435
*HSD3B2*	Hydroxy-delta-5-steroid dehydrogenase, 3β, and steroid delta-isomerase 2	Taqman	Hs00605123_m1
*NQO1*	NAD(P)H dehydrogenase, quinone 1	Taqman	Hs00168547_m1
*NQO2*	NAD(P)H dehydrogenase, quinone 2	Taqman	Hs00168552_m1
POLR2A *	DNA-directed RNA polymerase II subunit RPB1	TaqMan and SYBR Green	Hs00172187_m1 (TaqMan)
			Forward primer: CAAGTTCAACCAAGCCATTG (SYBR)
			Reverse primer: GTGGCAGGTTCTCCAAGG (SYBR)
RPLP0 *	60S acidic ribosomal protein P0	TaqMan and SYBR Green	Hs99999902_m1 (TaqMan)
			Forward primer: AATGTGGGCTCCAAGCAGAT (SYBR)
			Reverse primer: TTCTTGCCCATCAGCACCAC (SYBR)
*SLC10A6*	Solute carrier family 10 member 6	SYBR Green	Forward primer: TATGACAACCTGTTCCACCG
			Reverse primer: GAATGGTCAGGCACACAAGG
*SLC22A11*	Solute carrier family 22 member 11	SYBR Green	Forward primer: CTCACCTTCATCCTCCCCTG
			Reverse primer: CCATTGTCCAGCATGTGTGT
*SLC22A7*	Solute carrier family 22 member 7	SYBR Green	Forward primer: CCTCCAGAGTCCAAGGGTCT
			Reverse primer: ATGCTGCTCACCCACCAAAT
*SLC22A8*	Solute carrier family 22 member 8	SYBR Green	Forward primer: TACGCTGGTTGGTCTTGTCT
			Reverse primer: CTCCCTCTTCCTTCTTGCCA
*SLC22A9*	Solute carrier family 22 member 9	SYBR Green	Forward primer: CGGCTACCTATCTGACCCCA
			Reverse primer: TCTTGACGACTGTGCTTCCC
*SLC51A*	Organic solute transporter subunit alpha	SYBR Green	Forward primer: GCCCTTTCCAATACGCCTTC
			Reverse primer: TCTGCTGGGTCATAGATGCC
*SLC51B*	Organic solute transporter subunit beta	SYBR Green	Forward primer: GTGCTGTCAGTTTTCCTTCCG
			Reverse primer: TCATGTGTCTGGCTTAGGATGG
*SLCO1A2*	Solute carrier organic anion transporter family member 1A2	SYBR Green	Forward primer: GTTGGCATCATTCTGTGCAAATGTT
			Reverse primer: AACGAGTGTCAGTGGGAGTTATGAT
*SLCO1B1*	Solute carrier organic anion transporter family member 1B1	SYBR Green	Forward primer: CAAATTCTCATGTTTTACTG
			Reverse primer: GATTATTTCCATCATAGGTC
*SLCO1B3*	Solute carrier organic anion transporter family member 1B3	SYBR Green	Forward primer: TCCAGTCATTGGCTTTGCAC
			Reverse primer: TCCAACCCAACGAGAGTCCT
*SLCO1C1*	Solute carrier organic anion transporter family member 1C1	SYBR Green	Forward primer: CACACAGACTACCAAACACCC
			Reverse primer: TCACCATGCCGAACAGAGAA
*SLCO2B1*	Solute carrier organic anion transporter family member 2B1	SYBR Green	Forward primer: AGAGCCCTGTGTTCCATTCT
			Reverse primer: CTCTTGCTCCAGAAATGGCC
*SLCO3A1*	Solute carrier organic anion transporter family member 3A1	SYBR Green	Forward primer: CTACGACAATGTGGTCTAC
			Reverse primer: TTTTGATGTAGCGTTTATAG
*SLCO4A1*	Solute carrier organic anion transporter family member 4A1	SYBR Green	Forward primer: ATGCACCAGTTGAAGGACAG
			Reverse primer: AACAAGGTGGCAGCTTCTGAG
*SULT1A1*	Sulfotransferase family 1A, member 1	Taqman	Hs00738644_m1
*SULT1E1*	Sulfotransferase family 1E, estrogen-preferring, member 1	Taqman	Hs00193690_m1
*SLCO4C1*	Solute carrier organic anion transporter family member 4C1	SYBR Green	Forward primer: CCAGGAGCCCCAGAAGTC
			Reverse primer: AACTCGGACAGCGACAGTG
*STS*	Steroid sulfatase (microsomal), isozyme S	TaqMan	Hs00165853_m1
*SULT2A1*	Sulfotransferase family, cytosolic, 2A, dehydroepiandrosterone-preferring, member 1	Taqman	Hs00234219_m1
*SULT2B1*	Sulfotransferase family, cytosolic, 2B, member 1	Taqman	Hs00190268_m1
*UGT2B7*	UDP glucuronosyltransferase 2 family, polypeptide B7	Taqman	Hs00426592_m1

* Reference genes.

## Data Availability

The data presented in this study are available in the Supplementary Materials.

## Supplementary Materials

The following supporting information can be downloaded at: https://www.mdpi.com/article/10.3390/cancers14112583/s1, Table S1: R code used for hierarchical clustering; Table S2: Normalized mRNA values of evaluated genes in cell lines HIO-80, OVSAHO, Kuramochi, and COV362, normalized to the expression of *POLR2A* and *RPLP0*; Table S3: Evaluated transporters and HSD17B enzymes with K_M_ values for the transport of E1-S and transformations of E1 (to E2), E2 (to E1), or testosterone (to androstenedione); Table S4: Comparison of gene expressions in cell lines compared to each other, presented as fold regulation (FR); Table S5: Statistical analysis of LC-MS/MS results using Tukey’s test; Table S6: Levels of E1-S, E1, E2, and E2-S detected with LC-MS/MS after E1-S treatment (2.3, 8.5, and 85 nM) in cell lines HIO-80, OVSAHO, Kuramochi, and COV362; Table S7: Statistically significant differences in the gene expression in HGSOC tissues; Figure S1. Venn diagram representation of differential protein levels in the HGSOC subtypes.
