# Supplementary material for: Model Cell Lines and Tissues of Different HGSOC Subtypes Differ in Local Estrogen Biosynthesis
Source: Cancers (Basel). 2022 May 24;14(11):2583. doi: 10.3390/cancers14112583 (PMC9179372; doi:10.3390/cancers14112583)
Supplement: Supplementary file 1 [file cancers-14-02583-s001.zip › cancers-1640550-supplementary.pdf]

Supplementary Material: Model Cell Lines and Tissues of Different HGSOC Subtypes Differ in Local Estrogen Biosynthesis

Renata Pavlič, Marija Gjorgoska, Tea Lanišnik Rižner

Supplementary Table S1: R code used for hierarchical clustering.

```
> library("ComplexHeatmap")

> dim(all_genes_all_samples_log2)
> my_matrix <- as.matrix(all_genes_all_samples_log2[ ,c(2:50)])
> treatment_info <- data.frame(treatment = all_genes_all_samples_log2$SAMPLE_ID)
> my_matrix <- t(my_matrix)
> ht= Heatmap(my_matrix,
            cluster_columns = T,
            row_names_side = "left",
            row_dend_side = "left",
            row_dend_width = unit(2,"cm"),
            row_names_gp = gpar(fontsize = 10.5,fontface="italic"),
            column_names_gp = gpar(fontsize = 12),
            column_names_side = "bottom",
            clustering_distance_rows = "euclidean",
            clustering_method_rows = "ward.D",
            clustering_distance_columns = "euclidean",
            clustering_method_columns = "ward.D",
            show_column_names = T,
            width = unit(5, "cm"),

            heatmap_legend_param = list(title="mRNA,RSEM(log2_transformed)",
                                        title_gp=gpar(fontsize=10.5),
                                        labels_gp = gpar(fontsize = 10.5),
                                        title_position = "topcenter",
                                        legend_width=unit(3,"cm"),
                                        legend_direction="horizontal"))
> draw(ht,heatmap_legend_side="bottom")
```

Supplementary Table S2: Normalized mRNA values of evaluated genes in cell lines HIO-80, OVSAHO, Kuramochi, and COV362, normalized to the expression of *POLR2A* and *RPLP0*.

	**HIO-80**		**OVSAHO**		**Kuramochi**		**COV362**	
	**Normalized RNA x 10^{12}**	**SD**	**Normalized RNA x 10^{12}**	**SD**	**Normalized RNA x 10^{12}**	**SD**	**Normalized RNA x 10^{12}**	**SD**
ABCC1	255064.75	45182.04	209257.07	78746.21	248511.31	79832.68	401018.15	28250.79
ABCC11	0.00	0.00	0.00	0.00	0.00	0.00	0.00	0.00
ABCC4	20843.40	2667.48	62792.98	36864.07	10082.45	1629.39	12946.02	1896.82
ABCG2	109955.69	14418.96	4158.86	2348.07	719.29	431.79	103349.34	8274.44
AKR1C3	2877.78	227.62	230.08	56.12	4120.23	1317.78	80115.66	14717.16
COMT	534721.43	80311.08	153416.57	68543.36	380204.00	54176.75	163003.43	47587.76
CYP19A1	0.00	0.00	7.20	12.47	0.00	0.00	69.76	16.69
CYP1A1	4166.47	474.46	7186.04	5064.85	433.63	607.59	12378.74	2244.27
CYP1A2	0.00	0.00	0.00	0.00	0.00	0.00	3.81	6.60
CYP1B1	38741.33	4383.29	16452.79	7985.74	236635.26	74763.25	149195.88	20079.76
CYP3A5	280.96	37.78	0.00	0.00	0.00	0.00	0.00	0.00
CYP3A7	44.20	25.52	0.00	0.00	0.00	0.00	0.00	0.00
ESR1	1913.44	1047.10	704009.44	402645.14	1917.02	753.18	210087.08	16732.54
ESR2	1706.56	249.93	1435.86	554.46	1188.18	445.76	8078.48	1135.93
GPER v2	99.89	42.14	57.23	30.52	1355.94	195.53	1275.96	285.04
GPER v3, v4	10.38	5.08	30.38	14.54	968.09	286.23	375.13	131.23
GSTP1	1925678.85	155444.07	1423724.35	70951.57	1694448.48	261890.22	4080494.29	491527.46
HSD17B1	40.51	21.11	19.12	11.63	72.73	51.87	0.00	0.00
HSD17B10	248138.15	40563.89	512076.41	44864.03	188189.33	40254.61	240512.23	33896.12
HSD17B12	359565.55	48178.08	596976.04	66860.61	566296.21	18766.52	851396.97	42342.92
HSD17B14	1317.51	72.92	2093.44	750.22	7371.02	2620.78	9315.13	2824.36
HSD17B2	82.22	71.27	0.00	0.00	88.28	152.90	1157.18	240.92
HSD17B4	503264.73	43130.43	538450.29	45056.71	284167.02	18513.63	199467.36	13440.46
HSD17B7	452643.40	130628.04	167790.69	29333.10	289001.57	56357.43	431532.04	66633.63
HSD17B8	1935.81	342.39	3133.39	915.73	7123.08	1334.49	6347.56	486.06
HSD3B1	81.41	20.13	0.00	0.00	4.91	8.50	0.00	0.00
HSD3B2	0.00	0.00	8.44	8.29	10.24	9.34	0.00	0.00
NQO1	1801461.13	169802.00	261003.25	56251.58	362281.62	58851.87	3750066.73	1203603.02
NQO2	11041.48	1280.71	5374.98	1383.29	8766.55	2334.51	5359.44	212.16

SLC10A6	51.04	11.42	33.42	21.54	55.63	13.44	9.19	4.37
SLC22A11	1427.13	970.74	2308.47	2501.34	2112.59	605.71	3016.40	902.61
SLC22A7	85.72	27.56	91.23	118.20	32.51	28.19	65.02	26.06
SLC22A8	88.51	30.92	151.53	141.51	205.74	101.53	160.20	99.89
SLC22A9	101.23	16.49	621.62	616.89	274.66	59.96	361.09	81.62
SLC51A	1352.09	85.93	4255.89	3309.45	4142.97	148.02	2057.23	960.19
SLC51B	11.72	7.85	209.14	75.21	258.33	49.26	160.65	31.02
SLCO1A2	119.14	12.20	193.26	119.24	1270.41	728.11	8.20	8.85
SLCO1B1	0.00	0.00	0.00	0.00	0.00	0.00	0.00	0.00
SLCO1B3	1997.97	497.28	26.47	45.85	666.01	160.86	2764.72	600.05
SLCO1C1	4.01	6.95	23.06	23.48	103.24	71.03	37.97	31.89
SLCO2B1	688.74	182.35	186.85	255.56	975.76	606.90	9111.07	2200.90
SLCO3A1	204.47	181.81	784.03	801.76	189.13	170.94	994.42	449.22
SLCO4A1	1796.37	145.12	37951.27	21775.58	8393.78	1610.82	65542.01	7427.61
SLCO4C1	12270.28	3808.45	1410.05	452.87	2310.55	719.37	554.25	53.89
STS	119275.51	11839.59	38065.91	15348.03	60174.97	23066.40	108653.55	11717.34
SULT1A1	0.00	0.00	0.00	0.00	0.00	0.00	0.00	0.00
SULT1E1	70.36	22.86	51.27	20.64	929.30	264.22	1072.70	498.15
SULT2A1	0.00	0.00	0.00	0.00	0.00	0.00	7.72	13.37
SULT2B1	159.87	11.27	189.60	13.22	73.75	16.61	327.85	59.02
UGT2B7	0.00	0.00	0.00	0.00	0.00	0.00	0.00	0.00

Supplementary Table S3: Evaluated transporters and HSD17B enzymes with K_M values for the transport of E1-S and transformations of E1 (to E2), E2 (to E1), or testosterone (to androstenedione).

Genes	Proteins	K_M
ABCC1	MRP1	E1-S: 0.7-4.2 μM (summarized in Pavlič et al., 2021)
ABCC11	MRP8	E1-S: > 150 μM (Bortfeld et al., 2006)
ABCC4	MRP4	E1-S: NA
ABCG2	BCRP	E1-S: 6.8-16.6 μM (summarized in Pavlič et al., 2021)
HSD17B1	HSD17B1	E1: 0.9 μM (Puranen et al., 1997)
HSD17B10	HSD17B10	E2: NA<br>Testosterone: NA
HSD17B12	HSD17B12	E1: 3.5 μM (Luu-The et al., 2006)
HSD17B14	HSD17B14	E2: 5.6 μM (Lukacik et al., 2007)<br>Testosterone: 470 μM (Lukacik et al., 2007)
HSD17B2	HSD17B2	E2: 0.21 μM (Wu et al., 1993)<br>Testosterone: 0.39 μM (Wu et al., 1993)
HSD17B4	HSD17B4	E2: NA
HSD17B7	HSD17B7	E1: 3.25 μM (Törn et al., 2003)
HSD17B8	HSD17B8	E2: NA<br>Testosterone: NA
SLC10A6	SOAT	E1-S: 12 μM (Karakus et al., 2018)
SLC22A11	OAT4	E1-S: 1-22 μM (summarized in Pavlič et al., 2021)
SLC22A7	OAT2	E1-S: NA
SLC22A8	OAT3	E1-S: 2.2-21 μM (summarized in Pavlič et al., 2021)
SLC22A9	OAT7	E1-S: 8.7 μM (summarized in Pavlič et al., 2021)
SLC51A, SLC15B	OSTα/β	E1-S: 320 μM (summarized in Pavlič et al., 2021)
SLCO1A2	OATP1A2	E1-S: 7-59 μM (summarized in Pavlič et al., 2021)
SLCO1B1	OATP1B1	E1-S: 0.1-45 μM (summarized in Pavlič et al., 2021)
SLCO1B3	OATP1B3	E1-S: 5-58 μM (summarized in Pavlič et al., 2021)
SLCO1C1	OATP1C1	E1-S: NA
SLCO2B1	OATP2B1	E1-S: 1.6-21 μM (summarized in Pavlič et al., 2021)
SLCO3A1	OATP3A1	E1-S: NA
SLCO4A1	OATP4A1	E1-S: NA
SLCO4C1	OATP4C1	E1-S: 27 μM (summarized in Pavlič et al., 2021)

Pavlič et al., 2021, doi 10.3390/ijms22083819; Bortfeld et al., 2006, 10.1016/j.neuroscience.2005.10.025; Puranen et al., 1997, 10.1210/mend.11.1.9872; Luu-The et al., 2006, doi: 10.1210/me.2005-0058; Lukacik et al., 2007, doi: 10.1042/BJ20061319; Wu et al., 1993, PMID: 8099587; Törn et al., 2003, doi: 10.1016/s0006-291x(03)00694-6; Karakus et al., 2018, doi: 10.3389/fphar.2018.00941; NA, no data available.

Supplementary Table S4: Comparison of gene expressions in cell lines compared to each other, presented as fold regulation (FR).

	OVSAHO/HIO-80			Kuramochi/HIO-80			COV362/HIO-80			Kuramochi/OVSAHO			COV362/OVSAHO			COV362/Kuramochi		
	FR	p value		FR	p value		FR	p value		FR	p value		FR	p value		FR	p value	
ABCC1	-1.22	>0.9999	ns	-1.03	>0.9999	ns	1.57	0.5366	ns	1.19	>0.9999	ns	1.92	0.1412	ns	1.61	0.1887	ns
ABCC11			ns			ns			ns			ns			ns			ns
ABCC4	3.01	>0.9999	ns	-2.07	0.3255	ns	-1.61	>0.9999	ns	-6.23	0.0194	*	-4.85	0.1887	ns	1.28	>0.9999	ns
ABCG2	-26.44	0.6775	ns	-152.87	0.0552	ns	-1.06	>0.9999	ns	-5.78	>0.9999	ns	24.85	0.8462	ns	143.68	0.0764	ns
AKR1C3	-12.51	>0.9999	ns	1.43	>0.9999	ns	27.84	0.3255	ns	17.91	0.3255	ns	348.21	0.0134	*	19.44	>0.9999	ns
COMT	-3.49	0.0552	ns	-1.41	>0.9999	ns	-3.28	0.0764	ns	2.48	0.6775	ns	1.06	>0.9999	ns	-2.33	0.8462	ns
CYP19A1		>0.9999	ns		>0.9999	ns		0.0517	ns		>0.9999	ns	9.69	0.2597	ns		0.0517	ns
CYP1A1	1.72	>0.9999	ns	-9.61	>0.9999	ns	2.97	0.6775	ns	-16.57	0.4202	ns	1.72	>0.9999	ns	28.55	0.0194	*
CYP1A2		>0.9999	ns		>0.9999	ns		0.9438	ns		>0.9999	ns		0.9438	ns		0.9438	ns
CYP1B1	-2.35	>0.9999	ns	6.11	0.3255	ns	3.85	>0.9999	ns	14.38	0.0194	*	9.07	0.1887	ns	-1.59	>0.9999	ns
CYP3A5		0.0448	*		0.0448	*		0.0448	*		>0.9999	ns		>0.9999	ns		>0.9999	ns
CYP3A7		0.0448	*		0.0448	*		0.0448	*		>0.9999	ns		>0.9999	ns		>0.9999	ns
ESR1	367.93	0.0552	ns	1.00	>0.9999	ns	109.80	0.6775	ns	-367.24	0.0764	ns	-3.35	>0.9999	ns	109.59	0.8462	ns
ESR2	-1.19	>0.9999	ns	-1.44	>0.9999	ns	4.73	0.8462	ns	-1.21	>0.9999	ns	5.63	0.1887	ns	6.80	0.0764	ns
GPER v2	-1.75	>0.9999	ns	13.57	0.4202	ns	12.77	0.5366	ns	23.69	0.1045	ns	22.30	0.1412	ns	-1.06	>0.9999	ns
GPER v3, v4	2.93	>0.9999	ns	93.30	0.0134	*	36.15	0.2492	ns	31.86	0.2492	ns	12.35	>0.9999	ns	-2.58	>0.9999	ns
GSTP1	-1.35	0.5366	ns	-1.14	>0.9999	ns	2.12	>0.9999	ns	1.19	>0.9999	ns	2.87	0.0194	*	2.41	0.3255	ns
HSD17B1	-2.12	>0.9999	ns	1.80	>0.9999	ns		0.1816	ns	3.80	>0.9999	ns		0.8295	ns		0.0727	ns
HSD17B10	2.06	0.6775	ns	-1.32	>0.9999	ns	-1.03	>0.9999	ns	-2.72	0.0279	*	-2.13	0.5366	ns	1.28	>0.9999	ns
HSD17B12	1.66	0.5366	ns	1.57	>0.9999	ns	2.37	0.0134	*	-1.05	>0.9999	ns	1.43	>0.9999	ns	1.50	0.5366	ns
HSD17B14	1.59	>0.9999	ns	5.59	0.1412	ns	7.07	0.0279	*	3.52	>0.9999	ns	4.45	0.4202	ns	1.26	>0.9999	ns
HSD17B2		>0.9999	ns	1.07	>0.9999	ns	14.07	0.4781	ns		>0.9999	ns		0.0392	*	13.11	0.2394	ns
HSD17B4	1.07	>0.9999	ns	-1.77	>0.9999	ns	-2.52	0.1412	ns	-1.89	0.4202	ns	-2.70	0.0279	*	-1.42	>0.9999	ns
HSD17B7	-2.70	0.0764	ns	-1.57	>0.9999	ns	-1.05	>0.9999	ns	1.72	>0.9999	ns	2.57	0.0764	ns	1.49	>0.9999	ns
HSD17B8	1.62	>0.9999	ns	3.68	0.0552	ns	3.28	0.1412	ns	2.27	0.4202	ns	2.03	0.8462	ns	-1.12	>0.9999	ns
HSD3B1		0.0517	ns	-16.60	0.2597	ns		0.0517	ns		>0.9999	ns		>0.9999	ns		>0.9999	ns
HSD3B2		0.8300	ns		0.4792	ns		>0.9999	ns	1.21	>0.9999	ns		0.83	ns		0.4792	ns
NQO1	-6.90	0.3255	ns	-4.97	>0.9999	ns	2.08	>0.9999	ns	1.39	>0.9999	ns	14.37	0.0194	*	10.35	0.1887	ns
NQO2	-2.05	0.1412	ns	-1.26	>0.9999	ns	-2.06	0.0764	ns	1.63	>0.9999	ns	-1.00	>0.9999	ns	-1.64	0.6775	ns
SLC10A6	-1.53	>0.9999	ns	1.09	>0.9999	ns	-5.56	0.2492	ns	1.66	0.6775	ns	-3.64	>0.9999	ns	-6.06	0.0552	ns
SLC22A11	1.62	>0.9999	ns	1.48	>0.9999	ns	2.11	0.4202	ns	-1.09	>0.9999	ns	1.31	>0.9999	ns	1.43	>0.9999	ns
SLC22A7	1.06	>0.9999	ns	-2.64	0.3676	ns	-1.32	>0.9999	ns	-2.81	>0.9999	ns	-1.40	>0.9999	ns	2.00	>0.9999	ns
SLC22A8	1.71	>0.9999	ns	2.32	>0.9999	ns	1.81	>0.9999	ns	1.36	>0.9999	ns	1.06	>0.9999	ns	-1.28	>0.9999	ns
SLC22A9	6.14	0.1412	ns	2.71	0.6775	ns	3.57	0.1412	ns	-2.26	>0.9999	ns	-1.72	>0.9999	ns	1.31	>0.9999	ns

SLC51A	3.15	0.5366	ns	3.06	0.0764	ns	1.52	>0.9999	ns	-1.03	>0.9999	ns	-2.07	>0.9999	ns	-2.01	0.8462	ns
SLC51B	17.84	0.3255	ns	22.04	0.0279	*	13.71	>0.9999	ns	1.24	>0.9999	ns	-1.30	>0.9999	ns	-1.61	0.8462	ns
SLCO1A2	1.62	>0.9999	ns	10.66	0.5366	ns	-14.53	>0.9999	ns	6.57	>0.9999	ns	-23.58	0.5366	ns	-154.98	0.0134	*
SLCO1B1			ns			ns			ns			ns			ns			ns
SLCO1B3	-75.48	0.1869	ns	-3.00	>0.9999	ns	1.38	>0.9999	ns	25.16	>0.9999	ns	104.44	0.0191	*	4.15	0.3229	ns
SLCO1C1	5.75	>0.9999	ns	25.73	0.1354	ns	9.46	0.8295	ns	4.48	>0.9999	ns	1.65	>0.9999	ns	-2.72	>0.9999	ns
SLCO2B1	-3.69	>0.9999	ns	1.42	>0.9999	ns	13.23	0.5366	ns	5.22	0.8462	ns	48.76	0.0194	*	9.34	0.8462	ns
SLCO3A1	3.83	>0.9999	ns	-1.08	>0.9999	ns	4.86	0.4724	ns	-4.15	0.9374	ns	1.27	>0.9999	ns	5.26	0.2829	ns
SLCO4A1	21.13	0.1887	ns	4.67	>0.9999	ns	36.49	0.0194	*	-4.52	>0.9999	ns	1.73	>0.9999	ns	7.81	0.3255	ns
SLCO4C1	-8.70	0.3255	ns	-5.31	>0.9999	ns	-22.14	0.0134	*	1.64	>0.9999	ns	-2.54	>0.9999	ns	-4.17	0.3255	ns
STS	-3.13	0.0395	*	-1.98	0.1887	ns	-1.10	>0.9999	ns	1.58	>0.9999	ns	2.85	0.3255	ns	1.81	>0.9999	ns
SULT1A1			ns			ns			ns			ns			ns			ns
SULT1E1	-1.37	>0.9999	ns	13.21	0.4202	ns	15.25	0.3255	ns	18.12	0.1887	ns	20.92	0.1412	ns	1.15	>0.9999	ns
SULT2A1		>0.9999	ns		>0.9999	ns		0.9438	ns		>0.9999	ns		0.9438	ns		0.9438	ns
SULT2B1	1.19	>0.9999	ns	-2.17	>0.9999	ns	2.05	0.2492	ns	-2.57	0.2492	ns	1.73	>0.9999	ns	4.45	0.0134	*
UGT2B7			ns			ns			ns			ns			ns			ns

Statistical analysis: Kruskal-Wallis with Dunn's multiple comparisons test. *, $p < 0.05$; orange highlight, significantly down-regulated genes; blue highlight, significantly up-regulated genes.

Supplementary Table S5: Statistical analysis of LC-MS/MS results using Tukey's test. Formation of metabolites E1-S, E1, E2, and E2-S was compared in individual time points (8, 24, 48, 72 h) for all combinations of four cell lines (HIO-80, OVSAHO, Kuramochi, COV362).

**Formation of metabolites in cell lines**	**treatment**					
	**2.3 nM E1-S**		**8.5 nM E1-S**		**85 nM E1-S**	
	**p value**		**p value**		**p value**	
E1-S, 8 h						
HIO-80 / OVSAHO	0,1050	ns	0,9987	ns	0,9972	ns
HIO-80 / Kuramochi	0,0022	**	0,7244	ns	0,9916	ns
HIO-80 /COV362	<0,0001	****	0,5347	ns	0,3258	ns
OVSAHO / Kuramochi	0,9999	ns	0,8240	ns	0,9979	ns
OVSAHO / COV362	0,5187	ns	0,6351	ns	0,2117	ns
Kuramochi / COV362	0,0610	ns	0,8177	ns	0,5337	ns
E1-S, 24 h						
HIO-80 / OVSAHO	0,1560	ns	0,9092	ns	0,0426	*
HIO-80 / Kuramochi	0,7846	ns	0,3906	ns	0,4843	ns
HIO-80 /COV362	0,5619	ns	0,9996	ns	0,0203	*
OVSAHO / Kuramochi	0,0554	ns	0,0663	ns	0,3721	ns
OVSAHO / COV362	0,9349	ns	0,8565	ns	0,9602	ns
Kuramochi / COV362	0,7840	ns	0,4251	ns	0,1953	ns
E1-S, 48 h						
HIO-80 / OVSAHO	0,0193	*	0,8247	ns	0,2239	ns
HIO-80 / Kuramochi	0,0113	*	0,1968	ns	0,9966	ns
HIO-80 /COV362	0,6081	ns	0,9999	ns	0,1067	ns
OVSAHO / Kuramochi	>0,9999	ns	0,0238	*	0,5652	ns
OVSAHO / COV362	0,9488	ns	0,9488	ns	0,8844	ns
Kuramochi / COV362	0,9417	ns	0,4083	ns	0,3609	ns
E1-S, 72 h						
HIO-80 / OVSAHO	0,0478	*	0,1547	ns	0,0037	**
HIO-80 / Kuramochi	0,9820	ns	0,1631	ns	0,8106	ns
HIO-80 /COV362	0,9961	ns	0,6552	ns	0,3091	ns
OVSAHO / Kuramochi	0,1591	ns	0,0254	*	0,0695	ns
OVSAHO / COV362	0,1051	ns	0,0823	ns	0,0677	ns
Kuramochi / COV362	0,9710	ns	0,2007	ns	0,3791	ns
E1, 8 h						
HIO-80 / OVSAHO	0,5662	ns	0,1270	ns	0,3593	ns
HIO-80 / Kuramochi	0,4031	ns	0,8250	ns	0,0219	*
HIO-80 /COV362	0,1811	ns	0,9035	ns	0,1927	ns
OVSAHO / Kuramochi	0,9695	ns	0,1848	ns	0,9992	ns
OVSAHO / COV362	0,4819	ns	0,0740	ns	0,1785	ns
Kuramochi / COV362	0,7530	ns	0,1412	ns	0,0102	*
E1, 24 h						
HIO-80 / OVSAHO	0,9643	ns	0,1543	ns	0,2021	ns
HIO-80 / Kuramochi	0,5167	ns	0,9499	ns	0,0037	**
HIO-80 /COV362	0,9960	ns	0,9957	ns	0,0002	***
OVSAHO / Kuramochi	0,7009	ns	0,0310	*	0,9523	ns
OVSAHO / COV362	0,9610	ns	0,2657	ns	0,0227	*
Kuramochi / COV362	0,9810	ns	0,9160	ns	0,0003	***

E1, 48 h						
HIO-80 / OVSAHO	0,0266	*	0,0179	*	0,0517	ns
HIO-80 / Kuramochi	0,9544	ns	0,9959	ns	0,4031	ns
HIO-80 /COV362	0,2167	ns	0,1357	ns	0,0048	**
OVSAHO / Kuramochi	0,0895	ns	0,0194	*	0,3938	ns
OVSAHO / COV362	0,0103	*	0,0034	**	0,0086	**
Kuramochi / COV362	0,6133	ns	0,1430	ns	0,0490	*
E1, 72 h						
HIO-80 / OVSAHO	0,1089	ns	0,0489	*	0,0037	**
HIO-80 / Kuramochi	0,7891	ns	0,3593	ns	0,1464	ns
HIO-80 /COV362	0,3233	ns	0,0385	*	0,0092	**
OVSAHO / Kuramochi	0,7416	ns	0,9233	ns	0,0089	**
OVSAHO / COV362	0,0131	*	0,0291	*	0,0030	**
Kuramochi / COV362	0,2416	ns	0,3129	ns	0,0063	**
E2, 8 h						
HIO-80 / OVSAHO	0,3229	ns	0,1180	ns	0,7493	ns
HIO-80 / Kuramochi	0,9876	ns	0,4894	ns	0,1963	ns
HIO-80 /COV362	0,0199	*	0,0757	ns	0,0567	ns
OVSAHO / Kuramochi	0,9016	ns	0,0599	ns	0,3291	ns
OVSAHO / COV362	0,0550	ns	0,0458	*	0,0601	ns
Kuramochi / COV362	0,5661	ns	0,9796	ns	0,0747	ns
E2, 24 h						
HIO-80 / OVSAHO	0,8253	ns	0,8034	ns	0,0064	**
HIO-80 / Kuramochi	0,7025	ns	0,5926	ns	0,2710	ns
HIO-80 /COV362	0,7193	ns	0,4262	ns	0,0243	*
OVSAHO / Kuramochi	0,3040	ns	0,5116	ns	0,0372	*
OVSAHO / COV362	0,2973	ns	0,4411	ns	0,0024	**
Kuramochi / COV362	0,9970	ns	0,6111	ns	0,0055	**
E2, 48 h						
HIO-80 / OVSAHO	0,0505	ns	0,0136	*	0,0204	*
HIO-80 / Kuramochi	0,7013	ns	0,4600	ns	0,3337	ns
HIO-80 /COV362	0,6306	ns	0,0373	*	0,0050	**
OVSAHO / Kuramochi	0,9995	ns	0,0095	**	0,0358	*
OVSAHO / COV362	0,0082	**	0,0027	**	0,0105	*
Kuramochi / COV362	0,4878	ns	0,6257	ns	0,0772	ns
E2, 72 h						
HIO-80 / OVSAHO	0,0211	*	0,0359	*	0,0718	ns
HIO-80 / Kuramochi	0,6875	ns	0,3509	ns	0,0869	ns
HIO-80 /COV362	0,8899	ns	0,0053	**	0,0265	*
OVSAHO / Kuramochi	0,6357	ns	0,9891	ns	0,0927	ns
OVSAHO / COV362	0,0146	*	0,0200	*	0,0511	ns
Kuramochi / COV362	0,5398	ns	0,2813	ns	0,0012	**
E2-S, 8 h						
HIO-80 / OVSAHO	0,9447	ns	0,9874	ns	0,9791	ns
HIO-80 / Kuramochi	0,0583	ns	0,9713	ns	0,9303	ns
HIO-80 /COV362	0,0342	*	0,9819	ns	0,0392	*
OVSAHO / Kuramochi	0,2721	ns	0,8479	ns	0,8549	ns
OVSAHO / COV362	0,1908	ns	0,9972	ns	0,0316	*
Kuramochi / COV362	0,5083	ns	0,8399	ns	0,6593	ns

E2-S, 24 h						
HIO-80 / OVSAHO	0,8097	ns	0,0678	ns	0,0443	*
HIO-80 / Kuramochi	0,5220	ns	0,5073	ns	0,0835	ns
HIO-80 /COV362	0,1431	ns	0,6547	ns	0,0151	*
OVSAHO / Kuramochi	>0,9999	ns	0,5235	ns	0,9960	ns
OVSAHO / COV362	0,9224	ns	0,9975	ns	0,4927	ns
Kuramochi / COV362	0,5164	ns	0,8874	ns	0,4573	ns
E2-S, 48 h						
HIO-80 / OVSAHO	0,9237	ns	0,9686	ns	0,8938	ns
HIO-80 / Kuramochi	0,6336	ns	0,9315	ns	0,8619	ns
HIO-80 /COV362	0,4909	ns	0,8975	ns	0,1899	ns
OVSAHO / Kuramochi	0,9591	ns	0,9937	ns	0,9892	ns
OVSAHO / COV362	0,9064	ns	0,5749	ns	0,3162	ns
Kuramochi / COV362	0,9984	ns	0,5413	ns	0,6006	ns
E2-S, 72 h						
HIO-80 / OVSAHO	0,9808	ns	0,5826	ns	0,9034	ns
HIO-80 / Kuramochi	0,3509	ns	0,8669	ns	0,9876	ns
HIO-80 /COV362	0,2994	ns	0,4589	ns	0,0129	*
OVSAHO / Kuramochi	0,4176	ns	0,9943	ns	0,9892	ns
OVSAHO / COV362	0,3432	ns	0,2679	ns	0,6645	ns
Kuramochi / COV362	0,9991	ns	0,6389	ns	0,4080	ns

Statistical differences are highlighted in orange.

Supplementary Table S6: Levels of E1-S, E1, E2, and E2-S detected with LC-MS/MS after E1-S treatment (2.3, 8.5, and 85 nM) in cell lines HIO-80, OVSAHO, Kuramochi, and COV362.

Treatment with 2.3 nM E1-S

	Cell line							
	HIO-80		OVSAHO		Kuramochi		COV 362	
	mean	SD	mean	SD	mean	SD	mean	SD
**E1-S levels (ng/mL)**								
8 h	36.500	1.299	28.025	4.834	28.200	1.867	24.225	1.473
24 h	33.740	6.305	25.875	2.438	31.000	1.344	27.833	5.590
48 h	35.540	2.919	28.325	2.396	28.275	1.729	30.375	7.469
72 h	30.860	1.839	23.475	3.371	30.075	4.050	31.350	4.324
**E1 levels (ng/mL)**								
8 h	0.259	0.120	0.159	0.084	0.133	0.071	0.090	0.022
24 h	0.339	0.085	0.389	0.181	0.263	0.074	0.311	0.253
48 h	0.380	0.078	0.609	0.091	0.339	0.148	0.217	0.122
72 h	0.406	0.289	0.846	0.194	0.623	0.386	0.147	0.047
**E2 levels (ng/mL)**								
8 h	0.141	0.027	0.241	0.094	0.170	0.164	0.029	0.018
24 h	0.125	0.063	0.162	0.053	0.077	0.058	0.084	0.041
48 h	0.090	0.046	0.174	0.030	0.167	0.131	0.060	0.022
72 h	0.101	0.051	0.295	0.073	0.192	0.150	0.075	0.061
**E2-S levels (ng/mL)**								
8 h	1.985	0.303	2.198	0.727	1.353	0.196	1.193	0.079
24 h	2.286	0.751	1.683	1.011	1.708	0.448	1.320	0.238
48 h	2.400	0.943	2.013	0.935	1.728	0.706	1.658	0.523
72 h	2.712	1.034	2.493	0.726	1.768	0.466	1.730	0.317

Treatment with 8.5 nM E1-S

	Cell line							
	HIO-80		OVSAHO		Kuramochi		COV 362	
	mean	SD	mean	SD	mean	SD	mean	SD
**E1-S levels (ng/mL)**								
8 h	102.425	24.024	104.950	24.320	116.000	5.657	121.000	5.657
24 h	103.275	20.637	95.200	13.413	123.000	4.243	104.750	20.123
48 h	103.900	16.354	95.875	8.955	126.500	4.950	102.925	25.414
72 h	110.000	7.257	83.800	17.509	147.500	10.607	116.500	8.185
**E1 levels (ng/mL)**								
8 h	0.257	0.157	0.577	0.179	0.326	0.021	0.205	0.001
24 h	0.704	0.389	1.288	0.120	0.809	0.083	0.628	0.561
48 h	1.018	0.244	1.850	0.285	0.988	0.088	0.459	0.339
72 h	1.087	0.209	3.430	0.997	4.095	1.407	0.584	0.169
**E2 levels (ng/mL)**								
8 h	0.132	0.037	0.281	0.090	0.069	0.043	0.058	0.006
24 h	0.303	0.194	0.532	0.466	0.171	0.009	0.126	0.066
48 h	0.174	0.034	0.462	0.088	0.111	0.041	0.062	0.047

72 h	0.231	0.046	0.911	0.257	0.843	0.279	0.061	0.031
**E2-S levels (ng/mL)**								
8 h	5.855	1.652	5.595	0.169	6.380	1.344	5.535	0.474
24 h	6.160	0.130	5.435	0.359	5.815	0.247	5.305	1.391
48 h	6.515	1.961	7.003	1.037	7.205	0.926	5.598	1.799
72 h	7.405	1.130	9.403	2.730	8.870	2.588	6.173	1.092

Treatment with 85 nM E1-S

	**Cell line**							
	**HIO-80**		**OVSAHO**		**Kuramochi**		**COV 362**	
	mean	SD	mean	SD	mean	SD	mean	SD
**E1-S levels (ng/mL)**								
8 h	1237.500	160.702	1257.500	132.508	1287.500	324.178	1027.750	154.191
24 h	1367.500	131.498	1041.000	124.582	1215.000	151.987	1006.000	75.666
48 h	1205.000	128.712	1004.750	128.160	1180.000	185.203	936.000	140.359
72 h	1117.500	51.881	815.500	75.518	1208.500	197.101	1010.750	93.785
**E1 levels (ng/mL)**								
8 h	2.365	1.300	6.598	4.214	6.875	1.647	0.589	0.451
24 h	8.428	0.812	13.650	3.894	12.625	1.053	1.223	0.355
48 h	14.525	2.421	26.575	5.551	19.900	4.551	1.600	0.301
72 h	15.925	3.282	45.650	6.828	22.900	4.267	2.143	0.566
**E2 levels (ng/mL)**								
8 h	0.363	0.139	0.484	0.195	0.952	0.434	0.059	0.040
24 h	0.640	0.177	1.345	0.188	0.884	0.159	0.099	0.029
48 h	1.138	0.199	4.370	1.014	1.887	0.566	0.091	0.028
72 h	1.468	0.452	10.543	4.355	2.305	0.285	0.124	0.067
**E2-S levels (ng/mL)**								
8 h	70.375	6.990	72.325	7.384	64.900	17.216	54.200	4.166
24 h	77.400	7.006	60.550	6.283	61.625	7.695	52.875	8.045
48 h	68.100	7.517	64.600	6.611	62.533	10.589	51.600	11.474
72 h	68.650	4.015	64.200	12.686	66.775	11.215	56.400	2.936

Supplementary Table S7: Statistically significant differences in gene expression in HGSOC tissues.

Compared genes	Mean Diff.	Summary	Adjusted P Value
SLC10A6 vs. SLCO2B1	-899	***	<0.0001
SLC10A6 vs. SLCO3A1	-1348	***	<0.0001
SLC22A11 vs. SLCO2B1	-898	***	<0.0001
SLC22A11 vs. SLCO3A1	-1347	***	<0.0001
SLC22A7 vs. SLCO2B1	-900	***	<0.0001
SLC22A7 vs. SLCO3A1	-1349	***	<0.0001
SLC22A8 vs. SLCO2B1	-900	***	<0.0001
SLC22A8 vs. SLCO3A1	-1350	***	<0.0001
SLC22A9 vs. SLCO2B1	-900	***	<0.0001
SLC22A9 vs. SLCO3A1	-1350	***	<0.0001
SLCO1A2 vs. SLCO2B1	-840	***	<0.0001
SLCO1A2 vs. SLCO3A1	-1289	***	<0.0001
SLCO1B1 vs. SLCO2B1	-899	***	<0.0001
SLCO1B1 vs. SLCO3A1	-1348	***	<0.0001
SLCO1B3 vs. SLCO2B1	-858	***	<0.0001
SLCO1B3 vs. SLCO3A1	-1307	***	<0.0001
SLCO1C1 vs. SLCO2B1	-899	***	<0.0001
SLCO1C1 vs. SLCO3A1	-1348	***	<0.0001
SLCO2B1 vs. SLCO3A1	-449	***	<0.0001
SLCO2B1 vs. SLCO4A1	826	***	<0.0001
SLCO2B1 vs. SLCO4C1	876	***	<0.0001
SLCO3A1 vs. SLCO4A1	1275	***	<0.0001
SLCO3A1 vs. SLCO4C1	1325	***	<0.0001
ABCC1 vs. ABCC11	1570	***	<0.0001
ABCC1 vs. ABCC4	391	***	<0.0001
ABCC1 vs. ABCG2	1511	***	<0.0001
ABCC1 vs. SLC51A	1535	***	<0.0001
ABCC1 vs. SLC51B	1571	***	<0.0001
ABCC11 vs. ABCC4	-1179	***	<0.0001
ABCC4 vs. ABCG2	1120	***	<0.0001
ABCC4 vs. SLC51A	1143	***	<0.0001
ABCC4 vs. SLC51B	1179	***	<0.0001
HSD17B1 vs. HSD17B14	-145	**	0.0014
HSD17B1 vs. SULT2B1	150	***	0.0006
HSD17B12 vs. HSD17B4	157	***	0.0002
AKR1C3 vs. CYP19A1	267	***	<0.0001
AKR1C3 vs. HSD17B10	-1871	***	<0.0001
AKR1C3 vs. HSD17B12	-1610	***	<0.0001
AKR1C3 vs. HSD17B2	271	***	<0.0001
AKR1C3 vs. HSD17B4	-1453	***	<0.0001
AKR1C3 vs. HSD17B8	-203	***	<0.0001
AKR1C3 vs. HSD3B1	275	***	<0.0001
AKR1C3 vs. HSD3B2	266	***	<0.0001
AKR1C3 vs. SULT1E1	255	***	<0.0001
AKR1C3 vs. SULT2A1	270	***	<0.0001
AKR1C3 vs. SULT2B1	197	***	<0.0001
CYP19A1 vs. HSD17B1	-220	***	<0.0001

CYP19A1 vs. HSD17B10	-2138	***	<0.0001
CYP19A1 vs. HSD17B12	-1876	***	<0.0001
CYP19A1 vs. HSD17B14	-364	***	<0.0001
CYP19A1 vs. HSD17B4	-1720	***	<0.0001
CYP19A1 vs. HSD17B7	-257	***	<0.0001
CYP19A1 vs. HSD17B8	-469	***	<0.0001
CYP19A1 vs. STS	-306	***	<0.0001
HSD17B1 vs. HSD17B10	-1918	***	<0.0001
HSD17B1 vs. HSD17B12	-1657	***	<0.0001
HSD17B1 vs. HSD17B2	224	***	<0.0001
HSD17B1 vs. HSD17B4	-1500	***	<0.0001
HSD17B1 vs. HSD17B8	-250	***	<0.0001
HSD17B1 vs. HSD3B1	227	***	<0.0001
HSD17B1 vs. HSD3B2	219	***	<0.0001
HSD17B1 vs. SULT1E1	208	***	<0.0001
HSD17B1 vs. SULT2A1	223	***	<0.0001
HSD17B10 vs. HSD17B12	262	***	<0.0001
HSD17B10 vs. HSD17B14	1774	***	<0.0001
HSD17B10 vs. HSD17B2	2142	***	<0.0001
HSD17B10 vs. HSD17B4	418	***	<0.0001
HSD17B10 vs. HSD17B7	1881	***	<0.0001
HSD17B10 vs. HSD17B8	1669	***	<0.0001
HSD17B10 vs. HSD3B1	2146	***	<0.0001
HSD17B10 vs. HSD3B2	2137	***	<0.0001
HSD17B10 vs. STS	1832	***	<0.0001
HSD17B10 vs. SULT1E1	2127	***	<0.0001
HSD17B10 vs. SULT2A1	2141	***	<0.0001
HSD17B10 vs. SULT2B1	2069	***	<0.0001
HSD17B12 vs. HSD17B14	1512	***	<0.0001
HSD17B12 vs. HSD17B2	1880	***	<0.0001
HSD17B12 vs. HSD17B7	1620	***	<0.0001
HSD17B12 vs. HSD17B8	1407	***	<0.0001
HSD17B12 vs. HSD3B1	1884	***	<0.0001
HSD17B12 vs. HSD3B2	1876	***	<0.0001
HSD17B12 vs. STS	1570	***	<0.0001
HSD17B12 vs. SULT1E1	1865	***	<0.0001
HSD17B12 vs. SULT2A1	1880	***	<0.0001
HSD17B12 vs. SULT2B1	1807	***	<0.0001
HSD17B14 vs. HSD17B2	368	***	<0.0001
HSD17B14 vs. HSD17B4	-1356	***	<0.0001
HSD17B14 vs. HSD3B1	372	***	<0.0001
HSD17B14 vs. HSD3B2	364	***	<0.0001
HSD17B14 vs. SULT1E1	353	***	<0.0001
HSD17B14 vs. SULT2A1	368	***	<0.0001
HSD17B14 vs. SULT2B1	295	***	<0.0001
HSD17B2 vs. HSD17B4	-1724	***	<0.0001
HSD17B2 vs. HSD17B7	-261	***	<0.0001
HSD17B2 vs. HSD17B8	-473	***	<0.0001
HSD17B2 vs. STS	-310	***	<0.0001

HSD17B4 vs. HSD17B7	1463	***	<0.0001
HSD17B4 vs. HSD17B8	1251	***	<0.0001
HSD17B4 vs. HSD3B1	1728	***	<0.0001
HSD17B4 vs. HSD3B2	1719	***	<0.0001
HSD17B4 vs. STS	1414	***	<0.0001
HSD17B4 vs. SULT1E1	1708	***	<0.0001
HSD17B4 vs. SULT2A1	1723	***	<0.0001
HSD17B4 vs. SULT2B1	1651	***	<0.0001
HSD17B7 vs. HSD17B8	-213	***	<0.0001
HSD17B7 vs. HSD3B1	264	***	<0.0001
HSD17B7 vs. HSD3B2	256	***	<0.0001
HSD17B7 vs. SULT1E1	245	***	<0.0001
HSD17B7 vs. SULT2A1	260	***	<0.0001
HSD17B7 vs. SULT2B1	187	***	<0.0001
HSD17B8 vs. HSD3B1	477	***	<0.0001
HSD17B8 vs. HSD3B2	469	***	<0.0001
HSD17B8 vs. STS	163	***	<0.0001
HSD17B8 vs. SULT1E1	458	***	<0.0001
HSD17B8 vs. SULT2A1	472	***	<0.0001
HSD17B8 vs. SULT2B1	400	***	<0.0001
HSD3B1 vs. STS	-314	***	<0.0001
HSD3B2 vs. STS	-306	***	<0.0001
STS vs. SULT1E1	295	***	<0.0001
STS vs. SULT2A1	309	***	<0.0001
STS vs. SULT2B1	237	***	<0.0001
CYP1A2 vs. CYP1B1	-733	**	0.0011
CYP1B1 vs. CYP3A5	716	**	0.0017
CYP1B1 vs. SULT2B1	683	**	0.0041
COMT vs. CYP1B1	797	***	0.0002
CYP1A1 vs. CYP1B1	-751	***	0.0007
CYP1B1 vs. CYP3A7	758	***	0.0006
CYP1B1 vs. SULT1E1	741	***	0.0009
COMT vs. CYP1A1	1548	***	<0.0001
COMT vs. CYP1A2	1530	***	<0.0001
COMT vs. CYP3A5	1513	***	<0.0001
COMT vs. CYP3A7	1555	***	<0.0001
COMT vs. GSTP1	-12225	***	<0.0001
COMT vs. NQO2	1159	***	<0.0001
COMT vs. SULT1A1	1111	***	<0.0001
COMT vs. SULT1E1	1538	***	<0.0001
COMT vs. SULT2B1	1480	***	<0.0001
COMT vs. UGT2B7	1235	***	<0.0001
CYP1A1 vs. GSTP1	-13773	***	<0.0001
CYP1A1 vs. NQO1	-1843	***	<0.0001
CYP1A2 vs. GSTP1	-13755	***	<0.0001
CYP1A2 vs. NQO1	-1825	***	<0.0001
CYP1B1 vs. GSTP1	-13022	***	<0.0001
CYP1B1 vs. NQO1	-1092	***	<0.0001
CYP3A5 vs. GSTP1	-13738	***	<0.0001

CYP3A5 vs. NQO1	-1808	***	<0.0001
CYP3A7 vs. GSTP1	-13780	***	<0.0001
CYP3A7 vs. NQO1	-1850	***	<0.0001
GSTP1 vs. NQO1	11930	***	<0.0001
GSTP1 vs. NQO2	13384	***	<0.0001
GSTP1 vs. SULT1A1	13336	***	<0.0001
GSTP1 vs. SULT1E1	13763	***	<0.0001
GSTP1 vs. SULT2B1	13705	***	<0.0001
GSTP1 vs. UGT2B7	13460	***	<0.0001
NQO1 vs. NQO2	1454	***	<0.0001
NQO1 vs. SULT1A1	1406	***	<0.0001
NQO1 vs. SULT1E1	1833	***	<0.0001
NQO1 vs. SULT2B1	1775	***	<0.0001
NQO1 vs. UGT2B7	1530	***	<0.0001
ESR1 vs. ESR2	2743	***	<0.0001
ESR1 vs. GPER1	2721	***	<0.0001

Statistical analysis: One-way ANOVA with Bonferroni corrections.

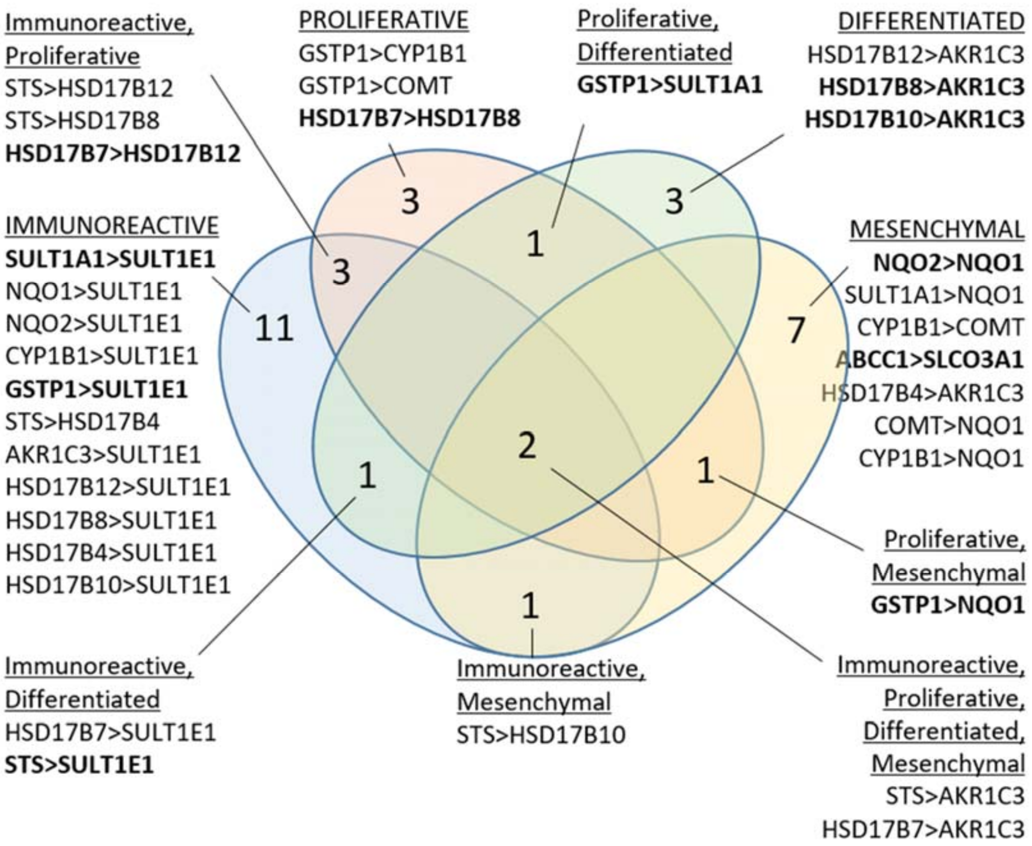

Supplementary Figure S1. Venn diagram representation of differential protein levels in HGSOC subtypes. Data from study Zhet al.t al, 2016 (TCGA Ovarian PNNL and JHU Proteome studies (IDs PDC000114, PDC000113)) were downloaded from the NCI, PDC server (https://pdc.cancer.gov) on 12. 1. 2022. Statistical analysis: One-way ANOVA with Bonferroni correction. »>« denotes »levels are higher than«.

Results indicate:

- in immunoreactive subtype: the referential transformation of E1-S to E1 (STS>SULT1E1, 2.3-fold),
- in proliferative subtype: the preferential activation of E1 to E2 (HSD17B7>HSD17B8, 1.3-fold; HSD17B7>HSD17B12 1.3-fold) and higher formation of glutathione conjugates compared to catechols (GSTP1>NQO1, 1.6-fold) and catechol sulphates (GSTP1>SULT1A1, 1.4-fold),
- in differentiated subtype: the preferential transformation of E1-S to E1 (STS>SULT1E1, 1.7-fold) and higher formation of glutathione conjugates compared to sulfated catechols (GSTP1>SULT1A1, 1.4-fold),
- in mesenchymal subtype: higher efflux of steroid precursors (ABCC1>SLCO3A1, 1.4-fold) and higher formation of 4-OH-estrogens (NQO2>NQO1, 1.7-fold) and glutathione conjugates (GSTP1>NQO1, 1.6-fold) compared to 2-OH-estrogens.
